# Critical Appraisal Tools for Evaluating Artificial Intelligence in Clinical Studies: Scoping Review

**DOI:** 10.2196/77110

**Published:** 2025-12-08

**Authors:** Juan B Cabello, Vicente Ruiz Garcia, Miguel Torralba, Miguel Maldonado Fernandez, Marimar Ubeda, Eukene Ansuategui, Luis Ramos-Ruperto, Jose I Emparanza, Iratxe Urreta, Maria Teresa Iglesias, Jose I Pijoan, Amanda Burls

**Affiliations:** 1 Critical Appraisal Skills Program Spain, C/ Enriqueta Elizaizin, 2, E 5, 7C, Alicante, 03007, Spain, 34 619669243; 2 Unidad de Hospitalización a Domicilio, Hospital Universitari i Politècnic La Fe, Valencia, Spain; 3 Servicio de Medicina Interna, Hospital Universitario de Guadalajara, Guadalajara, Spain; 4 Department of ENT, Hospital Vital Alvarez Buylla, Mieres, Spain; 5 Hospital Donostia, Donostia - San Sebastian, Spain; 6 Biblioteca virtual de salud de Euskadi, Vitoria, Spain; 7 Unidad de VIH, Medicina Interna, Hospital Universitario La Paz, Madrid, Spain; 8 Unidad de Epidemiologia Clínica e Investigación, CIBER-SP, Hospital Universitario Donostia, San Sebastian, Spain; 9 Instituto de Investigación Sanitaria Biobizkaia-Hospital Universitario Cruces, Bizkaia, Baracaldo, Spain; 10 City St George's, University of London, London, United Kingdom

**Keywords:** artificial intelligence, critical appraisal tools, scoping review, reporting guides, risk of bias

## Abstract

**Background:**

Health research that uses predictive and generative artificial intelligence (AI) is rapidly growing. As in traditional clinical studies, the way in which AI studies are conducted can introduce systematic errors. The translation of this AI evidence into clinical practice and research needs critical appraisal tools for clinical decision-makers and researchers.

**Objective:**

This study aimed to identify existing tools for the critical appraisal of clinical studies that use AI and to examine the concepts and domains these tools explore. The research question was framed using the Population-Concept-Context (PCC) framework. Population (P): AI clinical studies; Concept (C): tools for critical appraisal and associated constructs such as quality, reporting, validity, risk of bias, and applicability; and context (C): clinical practice. In addition, studies on bias classification and chatbot assessment were included.

**Methods:**

We searched medical and engineering databases (MEDLINE, Embase, CINAHL, PsycINFO, and IEEE) from inception to April 2024. We included clinical primary research with tools for critical appraisal. Classical reviews and systematic reviews were included in the first phase of screening and excluded in the secondary phase after identifying new tools by forward snowballing. We excluded nonhuman, computer, and mathematical research, and letters, opinion papers, and editorials. We used Rayyan (Qatar Computing Research Institute) for screening. Data extraction was done by two reviewers, and discrepancies were resolved through discussion. The protocol was previously registered in Open Science Framework. We adhered to the PRISMA-ScR (Preferred Reporting Items for Systematic reviews and Meta-Analyses extension for Scoping Reviews) and the PRISMA-S (PRISMA-Search) extension for reporting literature in systematic reviews.

**Results:**

We retrieved 4393 unique records for screening. After excluding 3803 records, 119 were selected for full-text screening. From these, 59 were excluded. After inclusion of 10 studies via other methods, a total of 70 records were finally included. We found 46 tools (26 guides for reporting AI studies, 16 tools for critical appraisal, 2 for study quality, and 2 for risk of bias). Nine papers focused on bias classification or mitigation. We found 15 chatbot assessment studies or systematic reviews of chatbot studies (6 and 9, respectively), which are a very heterogeneous group.

**Conclusions:**

The results picture a landscape of evidence tools where reporting tools predominate, followed by critical appraisal, and a few tools for risk of bias. The mismatch of bias in AI and epidemiology should be considered for critical appraisal, especially regarding fairness and bias mitigation in AI. Finally, chatbot assessment studies represent a vast and evolving field in which progress in design, reporting, and critical appraisal is necessary and urgent.

## Introduction

Much clinical research is unreliable because of systematic errors in the way the study was conducted or because the research findings are not generalizable to the context in which a decision is being made. When presented with research findings, it is very important, therefore, that clinicians and policymakers can assess the certainty of the evidence—that is, the level of confidence they can have that the estimated effect from a study or studies can be relied upon to support a particular decision or recommendation [1].

To help decision-makers decide whether research is trustworthy and applicable to their context, tools and checklists have been developed to critically appraise the validity, results, and relevance of clinical and health care studies. There are many different tools that are adapted for different study designs. In addition to critical appraisal tools, there are also tools to guide the reporting of studies, ensuring that all relevant information is transparently and accurately included in the “Methods” and “Results” sections. Many examples of reporting tools are provided on the EQUATOR Network Website.

As new technologies develop and study designs evolve, there is a need to update and develop new critical appraisal tools to look for potential biases and flaws in these designs and to ensure that there is guidance on how such studies should be reported transparently and fully.

The exponential growth of the use of artificial intelligence (AI) is among the most important innovations in health care and clinical studies design. The term “artificial intelligence” was coined in 1956 to refer to the activity of machines to mimic human intelligence or behavior [2]. Today, AI in health care encompasses a wide range of technologies and methods [3,4].

There are many types and definitions of AI, and these are expanding all the time. One broad categorization is generative versus predictive AI: the former creates new content, while the latter analyzes data to make predictions. Both are used in health care. Other common classifications of AI are outlined below. The different types of AI are not mutually exclusive but overlap and build upon one another.

Classic AI is a simple rule-based system with a defined structure that is programmed and does not learn.

Machine learning (ML) allows computers to learn from data and perform tasks without being explicitly programmed, improving with exposure to additional data.

Deep learning is a type of ML that uses multilayer algorithms to create an artificial neural network that can learn and make intelligent decisions on its own.

Artificial vision or computer vision uses algorithms that enable machines to capture, process, analyze, and interpret digital images and video.

Natural language processing is a type of ML that enables computers to understand and communicate with human language. It is used, for example, by chatbots (computer programs that simulate conversation with human end users).

Large language models (LLMs) are a further development of natural language processing that trains on large datasets to generate rather than analyze text. These form the basis of applications such as ChatGPT, launched by OpenAI in November 2022.

All these approaches are being used in clinical and health care settings, for example, to make diagnoses [5], identify cancers on imaging [6,7], assess prognosis [8], develop and test treatments, and create a diverse ecosystem of chatbots [7,9] that are currently a promising cutting edge in health care.

Just as traditional health research can have systematic errors that lead to biased or nongeneralizable results, so AI methods can introduce their own systematic errors during the design, data collection, training, or evaluation stages, which threaten the validity and reliability of AI models’ data analysis, findings, and conclusions. Such errors can arise from several different sources, including, but not limited to, flawed data, biased algorithms, and incorrect training.

Health care decision-makers, therefore, need to be able to critically appraise AI studies to detect these problems to be able to assess the certainty and relevance of the evidence they produce. Consequently, there is interest in the creation of new specific instruments or the adaptation of classic ones for the critical appraisal of AI studies [10,11].

The purpose of this paper was to undertake a scoping review to identify existing tools for critical appraisal of AI clinical studies and describe the concepts these tools address. We see this as the important first step toward being able to develop, evaluate, and recommend tools for future use.

## Methods

### Overview

This scoping review was designed and conducted according to the methodological framework of Levac et al [12] and the Joanna Briggs Institute (JBI [13]). We follow the PRISMA-ScR (Preferred Reporting Items for Systematic reviews and Meta-Analyses extension for Scoping Reviews [14]) and PRISMA-S (PRISMA-Search) for reporting literature searches in systematic reviews [15].

We used the PCC framework for scoping reviews [13]. Definitions of each element are given below under the review question.

The protocol was registered on the Open Science Framework on April 18, 2024 [16]. Amendments to the protocol are documented in this paper and in the protocol.

### PCC Definitions

#### Population

Existing tools to assess AI clinical studies. We included any type of study design and any clinical objective: diagnostic, prognostic, prediction rules, or decision-making systems. We included both predictive and generative AI.

#### Concepts

Studies describing tools for critical appraisal and associated constructs (completeness of reporting, validity of study, quality of study, risk of bias, and applicability), whether or not they had been formally evaluated. Modifications or adaptations of original tools were accepted. Studies focusing on a comprehensive approach to bias in AI and fairness, understood as the bioethical consequences of bias in AI clinical studies, were also included. Chatbot assessment studies, including primary research and systematic reviews, were included if they focused on clinical activities (diagnosis, prognosis, treatment, prevention, recommendations, or clinical decisions) and did not meet exclusion criteria (Table 1).

**Table 1.** Inclusion and exclusion criteria.

Inclusion criteria	Exclusion criteria
Any type of study, describing tools for critical appraisal and associated constructs with any design and any clinical objective: diagnostic, prognostic, prediction rules, or decision-making systems. Studies focused on clinicians and clinical centers and clinical activities both in and out of hospitals. Published protocols were accepted.	Studies on animals, nonhuman studies (specimens), studies focused on engineering, development of models, algorithms, or analysis of their mathematical properties, as well as artificial intelligence (AI) studies aimed at increasing image resolution or anatomical amplification, virtual reality, or simulations.
We included both predictive and generative AI.	Letters to the editor and opinion papers. Editorials were excluded, except if they included guidelines for reporting or reading AI studies. Experimental studies were excluded.
Studies focusing on a comprehensive approach to bias in AI and fairness, understood as the bioethical consequences of bias in AI clinical studies.	—^a^
Chatbot assessment studies, primary research, and systematic reviews focused on clinical activities (diagnosis, prognosis, treatment, prevention, recommendations, or clinical decisions).	—
For chatbot studies only, we accepted lists of questions, clinical scenarios, or vignettes used in initial chatbot performance assessments. We were flexible in these studies.	—
Classical reviews, systematic reviews, and congress abstracts describing or using AI critical appraisal tools were all included in the initial screening. Those that focused on AI biases or bias mitigation were included. The others were reviewed to identify any AI tools used. If they used AI tools, these tools were included in the review, but the systematic review itself was excluded.	—

a Not available.

#### Context

We focused on clinicians and clinical centers, and clinical activities both in and out of hospitals. Other clinical research or paraclinical areas were not included.

### Review Question

Three questions are addressed in this review:

#### Primary Question

What tools exist for critical appraisal of studies on AI in the clinical setting, and what constructs do they address (relevance of the question, completeness of reporting, validity of study, quality of study, risk of bias, and applicability)? After reflection and discussion within the group, we made an amendment to the protocol to change the specified focus on critical appraisal, since this encompassed all the associated constructs and was more suitable for the clinical setting (the quality of the study or risk of bias being more specific and more relevant for systematic reviews).

#### Subquestions

We anticipated 2 additional questions that would provide a more comprehensive evidence map of AI critical appraisal tools.

1. Concepts: all the above-mentioned constructs need to be adapted for the AI context, as AI studies may have different biases compared with classical epidemiological studies. Therefore, we sought to identify papers that focused on comprehensive reflections, catalogs, or glossaries of bias classification or bias mitigation in AI clinical studies.
2. Population: Because the upsurge in chatbot assessment studies in clinical research is so recent, we thought it was unlikely that we would find specific tools to assess their quality. Therefore, we looked at how the risk of bias is assessed in systematic reviews of these studies.

### Search Strategy

We searched the following electronic databases from inception to April 2024: MEDLINE, Embase, CINAHL, PsycINFO, and IEEE Advancing Technology for Humanity. Two scientific information specialists undertook the searches independently. Their results were compared, and the search was refined. The terms used were as follows:

(“artificial intelligence” OR “machine learning” OR “deep learning” OR “large language model” OR “computer vision” OR “artificial intelligence Chatbot” OR “ChatGPT”) AND (“risk assessment” OR “Bias” OR “quality assessment” OR “statistical bias” OR “reproducibility” OR “internal validity” OR “external validity” OR “critical appraisal” OR “reporting guideline” OR “checklist” OR “toolkit” OR “tools”). No language limitations were used.

We searched the following registries: PROSPERO [17], Open Science Framework [18], and the Research Registry [19].

We searched the EQUATOR Network [20] for reporting guidelines using the terms “artificial intelligence” OR “machine learning” OR “deep learning” AND “reporting guidelines.”

We tracked citations from the systematic reviews of tools identified in the first phase of screening (snowballing). Finally, we incorporated some papers recommended by experts.

We used Zotero (Sean Takats) as the main tool for managing references. A complete description of the process, including search dates, is available in Checklist 1 (checklist of PRISMA Searching).

For chatbot assessment studies, we used a free-text–based strategy using synonyms and truncations, because these are not yet Medical Subject Headings (MeSH) terms, so controlled language could not be used.

### Source of Evidence Selection

All searches were merged into a file and exported to Rayyan for screening. Duplicate documents identified by Rayyan were reviewed by an information specialist, and duplicates were removed.

#### First Phase (Screening by Title and Abstract)

We divided the retrieved papers into three randomized samples. Three groups of two researchers rated their allotted samples independently and in a blinded way. Disagreements flagged by Rayyan were resolved by discussion and consensus within each group session first, and then in a general session among groups. Two facilitators, not involved in the initial ratings, took part in all discussions (both within and among groups) to resolve disagreements and ensure consistent criteria across groups.

#### Second Phase (Full-Text Screening)

The selected set of references was rated in Rayyan by 2 groups of researchers working independently. Inconsistencies (between and within groups) were identified and resolved by discussion and consensus in a common session with the help of 2 facilitators. The exclusions during full-text screening and their reasons were recorded.

The AI tools identified from systematic review papers were included in “Identification via Other methods” (citation searching from systematic review of AI studies). These studies, and those obtained from the EQUATOR Network library and experts and organizations, were cross-referenced with the studies remaining after full-text screening for duplicates.

### Data Extraction

#### Main Question

We constructed and piloted a data template, informed by JBI [21], which included editorial data such as author, year, associated domains, main question, and associated constructs, as well as other features such as clinical use, practical conditions, object of the tool, methodological characteristics, number of items, and method for developing the tool.

The first version of the template was tested by 2 researchers on a set of 10 included papers. The data template was refined, when necessary, in an iterative process. After modifications, the final version of the template was piloted on another set of 10 included papers. The final version of the template is available in Multimedia Appendix 1.

The data were entered into Excel (Microsoft) independently by 2 researchers. Data inconsistencies were identified and resolved by discussion and consensus with a third reviewer.

#### Subquestions

For bias and bias mitigation, the following data were extracted independently by 2 researchers: author date, title, bias classification, bias mitigation, and free comments. The consistency of data and qualitative details was discussed, and agreement was reached by consensus with a third rater.

For chatbot assessment studies, a template was designed, piloted, and modified. The final version (refer to Multimedia Appendix 1) included author, year, topic, title, PICO (Population/Patient/Problem, Intervention, Comparison, and Outcome), risk-of-bias tools used, and open comments.

Data extraction for chatbot studies used a hybrid approach, combining the active involvement of a researcher with a fully supervised ChatGPT–retrieval-augmented generation model. This strategy was adopted given the predictable heterogeneity of chatbot interventions, with the aim of enhancing the clarity and reproducibility of extracted data. ChatGPT-4o was used to assist in drafting and refining the extraction tables, but all outputs were independently reviewed by 2 authors against the original papers. Discrepancies were reviewed and resolved through consensus. No sensitive data were exposed. To promote transparency and reproducibility, the exact prompts used in the retrieval-augmented generation process are shown in Multimedia Appendix 2.

## Results

### Search Result

We identified 4392 records from databases and registries. After eliminating 470 duplicates, 3922 records were screened by title and abstract, and 3803 were excluded. The remaining 119 underwent full-text screening, and 59 were excluded. The reasons for exclusion were as follows: 50 were systematic reviews, 7 studies met the exclusion criteria, and 2 did not meet the inclusion criteria. Full details are available in Multimedia Appendix 3 (exclusions after full-text screening). Of the 50 systematic reviews, 42 used specific AI tools to assess the quality of the studies, and the tools retrieved were incorporated into “records identified via other methods.”

**Figure 1.**
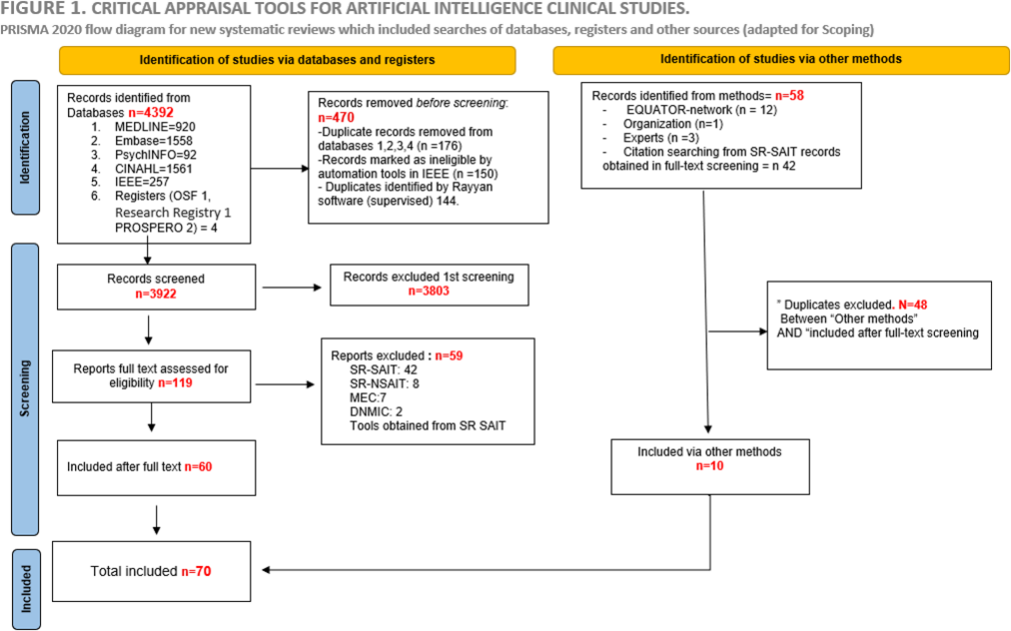
PRISMA (Preferred Reporting Items for Systematic reviews and Meta-Analyses) 2020 flow diagram adapted for scoping reviews.

Twelve studies were identified in the EQUATOR Network library, and 4 additional studies were obtained from experts and organizations; therefore, there were 58 records identified via other methods. Forty-eight of these were already captured in the 60 included studies from the search of electronic databases, leaving 10 additional studies to be included. Thus, a total of 70 studies were included in this review (refer to Figure 1).

### Characteristics of Included Studies

Of the 70 retrieved studies, 46 focused on the main question of the review: tools for critical appraisal and related constructs. The general characteristics of these studies are shown in Table 1 and Table 2. Nine papers were relevant to our second question and focusing on AI bias classification or bias mitigation (refer to Table 3). We found 15 chatbot assessment studies (6 were primary research studies and 9 were systematic reviews). The main characteristics of both types are shown in Table 4 and Table 5, respectively.

**Table 2.** Tools for critical appraisal and related constructs.

Author, year	Name of tool	Clinical aim	Clinical area or specialty	No of items	Setting/context of use	Construct	Design
Luo et al, 2016 [22]	Luo	CA/ML/AI^a^	Clinical and research use	56	Diagnosis/prognosis/treatment	Critical appraisal	Partially collaborative
Lambin et al, 2017 [23]	Radiomics Quality Score (RQS)	Diagnosis/progn/treatment	Radiology	36	Diagnosis/prognosis/treatment	Reporting	Experts
Qiao, 2019 [24]	Qiao	Diagnosis	Clinical and research use	20	Diagnosis/prognosis/treatment	Critical appraisal	Experts
Liu et al, 2019 [25]	Liu	CA/ML/AI	Clinical and research use	3	Diagnosis/prognosis/treatment	Critical appraisal	Experts
Vollmer et al, 2019 [26]	TREE	CA/ML/AI	Clinical and research use	20	Diagnosis/prognosis/treatment	Critical appraisal	Partially collaborative
Cruz Rivera et al, 2020 [27]	SPIRIT-AI^b^	Treatment	Clinical and research use	15	Diagnosis/prognosis/treatment	Reporting	Comprehensive
Faes et al, 2020 [28]	Faes	Diagnosis/Progn/Treat	Clinical and research use		Diagnosis/prognosis/treatment	Critical appraisal	Experts
Hernandez-Boussard et al, 2020 [29]	MINIMAR^c^	CA/ML/AI	Clinical and research use	21	Social (identifying or mitigating algorithmic bias)	Reporting	Experts
Liu et al, 2020 [30]	CONSORT-AI^d^	Treatment	Clinical and research use	13	Diagnosis/prognosis/treatment	Reporting	Comprehensive
Mongan et al, 2020 [31]	CLAIM^e^	Diagnosis	Radiology	42	Diagnosis/prognosis/treatment	Reporting	Partially collaborative
Norgeot et al, 2020 [32]	MI-CLAIM^f^	CA/ML/AI	Clinical and research use	22	Social (identifying or mitigating algorithmic bias)	Reporting	Experts
Sengupta et al, 2020 [33]	PRIME^g^	Diagnosis	Radiology	28	Diagnosis/prognosis/treatment	Reporting	Experts
Stevens et al, 2020 [34]	Stevens	CA/ML/AI	Clinical and research use		Social (identifying or mitigating algorithmic bias)	Reporting	Experts
Cabitza and Campagner, 2021 [35]	IJMEDI checklist	CA/ML/AI	Clinical and research use	30	Preclinical and clinical studies:	Reporting	Experts
El Naqua et al, 2021 [36]	CLAMP	CA/ML/AI	Radiation oncology	26	Preclinical and clinical studies:	Reporting	Experts
Kwong et al, 2021 [37]	STREAM-URO^h^	Prognosis	Urology	26	Diagnosis/prognosis/treatment	Reporting	Comprehensive
Meshaka et al, 2021 [38]	CLAIM-Pediatrics Rx	Diagnosis	Radiology	42	Diagnosis/prognosis/treatment	Reporting	Experts
Olczak et al, 2021 [39]	CAIR^i^	Diagnosis	Traumatology	36	Diagnosis/prognosis/treatment	Reporting	Experts
Schwendicke et al, 2021 [40]	Schwendicke	CA/ML/AI	Oral health	31	Preclinical studies (ML)	Reporting	Comprehensive
Sounderajah et al, 2021 [41]	QUADAS-AI^j^	Diagnosis	Clinical and research use	—	Preclinical studies (ML)	Risk of bias	Experts
Sounderajah et al, 2021 [42]	STARD-AI^k^	Diagnosis	Clinical and research use	—	Diagnosis/prognosis/treatment	Reporting	Comprehensive design
Vinny et al, 2021 [43]	Vinny	Diagnosis	Radiology	14	Diagnosis/prognosis/treatment	Critical appraisal	Experts
Collins et al, 2021 [44]	PROBAST-AI^l^	Prognosis	Clinical and research use	—	Diagnosis/prognosis/treatment	Risk of bias	Comprehensive
Al-Zaiti et al, 2022 [45]	ROBUST-ML^m^	Diagnosis/Progn/Treat	Clinical and research use	30	Diagnosis/prognosis/treatment	Critical appraisal	Comprehensive
Daneshjou et al, 2022 [46]	CLEAR/DERM^n^	Diagnosis	Dermatology	25	Clinical use of diagnosis/prognosis/treatment	Reporting	Comprehensive
Haller et al, 2022 [47]	R-AI-DIOLOGY	CA/ML/AI	Radiology	15	Preclinical and clinical studies	Critical appraisal	Experts
Jha et al, 2022 [48]	RELIANCE^o^ (CLAIM)	Diagnosis	Radiology	—	Diagnosis/prognosis/treatment	Critical appraisal	Comprehensive design
Padula et al, 2022 [49]	PALISADE^p^	CA/ML/AI	Clinical and research use	8	Social (identifying or mitigating algorithmic bias)	Critical appraisal	Experts
Van Smeden et al, 2022 [50]	Van-Smeden	Prognosis	Cardiology	12	Diagnosis/prognosis/treatment	Reporting	Experts
Vasey et al, 2022 [51]	DECIDE AI^q^	CA/ML/AI	Clinical and research use	27	Clinical evaluation of decision support systems	Reporting	Comprehensive
Jones et al, 2022 [52]	Jones	Prognosis/diagnosis	Dermatology	19	Diagnosis/prognosis/treatment	Critical appraisal	Comprehensive
Cabello, 2022 [53]	CASPE-AI^r^	CA/ML/AI	Clinical and research use	10	Diagnosis/prognosis/treatment	Critical appraisal	Experts
Cacciamani et al, 2023 [54]	PRISMA^s^-AI	Diagnosis/Progn/Treat	Clinical and research use	—	Preclinical and clinical studies	Reporting	Comprehensive
Alberich et al, 2023 [55]	MAIC-10^t^	Diagnosis	Radiology	10	Social (identifying or mitigating algorithmic bias)	Reporting	Partially collaborative
Kocak et al, 2023 [56]	CLEAR^u^	CA/ML/AI	Clinical and research use	58	Preclinical studies (ML)	Reporting	Comprehensive
Kwong et al, 2023 [57]	APPRAISE-AI	CA/ML/AI	Clinical and research use	24	Preclinical and clinical studies	Critical appraisal	Comprehensive
Park et al, 2023 [58]	Park	Diagnosis	Radiology	10	Diagnosis/prognosis/treatment	Reporting	Experts
FDA, 2023 [59]	FDA^v^	CA/ML/AI	Clinical and research use	10	Other	Critical appraisal	Experts
Collins et al, 2024 [60]	TRIPOD-AI^w^	Prognosis	Clinical and research use	52	Diagnosis/prognosis/treatment	Reporting	Comprehensive
Du Toit et al, 2023 [61]	HUMANE^x^	Prognosis/Diagnosis	Clinical and research use	55	Clinical use of diagnosis/prognosis/treatment	Critical appraisal	Partially Collaborative
Cote and Lubowitz, 2024 [62]	Cote	CA/ML/AI	Traumatology	—	Preclinical and clinical studies	Reporting	Experts
Kocak et al, 2024 [63]	METRICS^y^	CA/ML/AI	Radiology	30	Social (identifying or mitigating algorithmic bias)	Quality	Comprehensive
Lekadir et al, 2024 [64]	FUTURE-AI^z^	CA/ML/AI	Radiology	55	Preclinical studies (ML)	Reporting	Experts
Scott et al, 2024 [65]	Scott	CA/ML/AI	Clinical and research use	10	Diagnosis/prognosis/treatment	Critical appraisal	Experts
Vaira et al, 2024 [66]	QUAMAI^aa^ (ChatGPT; OpenAI)	Chatbot study	Clinical and research use	—	Diagnosis/prognosis/treatment	Quality	Comprehensive
CHART Collabor, 2024 [67]	CHART^ab^ (chatbots)	Chatbot study	Clinical and research use	—	Preclinical and clinical studies:	Reporting	Partially collaborative

a AI: artificial intelligence.

b SPIRIT-AI: Standard Protocol Items: Recommendations for Interventional Trials involving Artificial Intelligence.

c MINIMAR: Minimum Information for Medical AI Reporting.

d CONSORT-AI: Consolidated Standards of Reporting Trials extension for Artificial Intelligence.

e CLAIM: Checklist for Artificial Intelligence in Medical Imaging.

f MI-CLAIM: Minimum Information for Medical Artificial Intelligence Reporting.

g PRIME: Proposed Requirements for Cardiovascular Imaging Related MI Evaluation.

h STREAM-URO: Standardized Reporting of Machine Learning Applications in Urology.

i CAIR: Clinical Artificial Intelligence Research.

j QUADAS-AI: Quality Assessment of Diagnostic Accuracy Studies for Artificial Intelligence.

k STARD-AI: Standards for Reporting of Diagnostic Accuracy Studies for Artificial Intelligence.

l PROBAST-AI: Prediction model Risk Of Bias Assessment Tool for AI studies.

m ROBUST-ML: Ruling Out Bias Using Standard Tools in Machine Learning.

n CLEARDERM: Checklist for Evaluation of Image-Based Artificial Intelligence (AI) Algorithm Reports in Dermatology.

o RELAINCE (Recommendations for Evaluation of AI for Nuclear Medicine).

p PALISADE: Purpose, Appropriateness, Limitations, Implementation, Sensitivity and Specificity, Algorithm characteristics, Data characteristics, and Explainability.

q DECIDE AI: Developmental and Exploratory Clinical Investigations of Decision Support Systems Driven by Artificial Intelligence.

r CASPE-AI: CRITICAL APPRAISAL SKILLS PROGRAM ESPAÑA-Artificial Intelligence.

s PRISMA: Preferred Reporting Items for Systematic reviews and Meta-Analyses.

t MAIC-10 (Must AI Criteria-10).

u CLEAR: CheckList for EvaluAtion of Radiomics research

v FDA: Food and Drug Administration.

w TRIPOD-AI: Transparent Reporting of a multivariable prediction model for Individual Prognosis Or Diagnosis using Artificial Intelligence.

x HUMANE: Harmonious Understanding of Machine Learning Analytics Network.

y METRICS: Methodological Radiomics Score.

z FUTURE-AI: Fairness, Universality, Traceability, Usability, Robustness, and Explainability.

aa QUAMAI: Quality Analysis of Medical Artificial Intelligence.

ab CHART: Chatbot Assessment Reporting Tool.

**Table 3.** Bias classification and bias mitigation papers.

Author	Year	Title	Bias classification	Bias mitigation	Comments
Brault and Saxena [68]	2021	For a critical appraisal of artificial intelligence in health care: the problem of bias in mHealth^a^	Describes different steps where bias can be introduced during data collection, manipulation, or processing.	No information about this topic	Uses examples from contemporary use of mHealth apps
Feltcher et al [69]	2021	Addressing fairness, bias, and appropriate use of artificial intelligence and ML^b^ in global health	Systematic bias, sampling bias, and socioeconomic status bias.	Solutions to mitigate biases in all stages of algorithm development (sampling, regularization constraints, cost functions, and adversarial learning algorithms)	Uses an example of creating a model for diagnosing lung disease in primary care
Mehrabi et al [70]	2022	A survey on bias and fairness in ML	Bias from data to algorithm (measurement bias, omitted variable bias, representation bias, aggregation bias, sampling bias, longitudinal data fallacy, and linking bias); Bias from algorithm to user (algorithmic bias, user interaction bias, popularity bias, emergent bias, and evaluation bias); and bias from user to data (historical bias, population bias, and self-selection bias).	Provides a synthesis of fairness definitions and a fair classification with a causal reflection about unfairness. In addition, it includes a comparison of different mitigation algorithms.	Includes datasets for fairness research.
Swartz et al [71]	2022	Towards a standard for identifying and managing bias in artificial intelligence	Systemic, statistical, and human biases.	Outlines 3 major challenges to mitigating bias: datasets, testing and evaluation, and human factors.	Presents preliminary guidance to address bias
Xu et al [72]	2022	Algorithmic fairness in computational medicine	Computational bias (selection bias, attrition bias, publication bias, measurement bias, and algorithm bias).	Mitigation at preprocessing (demonstration and reweighting), internal processing (debiasing and adversarial learning), and postprocessing (matched odds and calibrated matched odds)	Summarizes available software libraries and tools for bias assessment and mitigation
Saint James Aquino [73]	2023	Making decisions: bias in artificial intelligence and data-driven diagnostic tools	Algorithmic bias	No information about this topic	—^c^
Park and Hu [74]	2023	Bias in artificial intelligence	Bias in data generation (data collection or determination of results), bias in model training, testing, and validation (model selection or treatment of missing values), and bias in model interpretation and application (acceptance or health literacy).	Preprocessing (reweighting), internal processing (reducing influence of a variable in the learning process), and postprocessing (adjusting the results in a post hoc manner). Also, discuss nonalgorithmic bias mitigation such as patient demographic distribution between training data and target population.	Includes a figure with the stages of artificial intelligence application development and associated biases.
Perez-Downes et al [75]	2024	Mitigating bias in clinical ML models	Algorithmic bias	Mitigation across domains: inclusivity (ensuring women and racial/ethnic minority groups are adequately represented in training datasets), specificity (ensuring that appropriate and specific training targets are selected when developing models), transparency (ensuring standard reporting to include information regarding training data, model annotation, and interpretability), validation (conducting rigorous testing/auditing), validation studies (internal and external), and clinical trials as appropriate before deploying ML^b^ models for use in clinical care.	Includes a figure illustrating a framework for mitigating bias, a figure with ethical challenges in ML for clinical research and practice, and examples of current applications of ML in clinical medicine.
Flores et al [76]	2024	Addressing bias in artificial intelligence for public health surveillance	Algorithmic bias resulting from data collection, labeling, and modeling of natural language processing (NLP)	The implementation of open collaboration, auditing processes, and the development of guidelines.	—

a mHealth: mobile health.

b ML: machine learning.

c Not available.

**Table 4.** Chatbot assessment studies (primary research).

Author	Year	Topic	Population	Intervention	Gold standard / Comparison	Outcome	Type of chatbots	Reporting
Yeo et al [77]	2023	Assessment of ChatGPT’s accuracy and consistency in answering questions	Set of questions related to cirrhosis and hepatocellular carcinoma (HCC).	ChatGPT responses	Compared to medical experts’ responses and guidelines.	Accuracy, consistency. ChatGPT showed good performance but lacked specificity in regional recommendations.	LLM^a^ (GPT-3.5–based chatbot trained until 2021).	Addressed hallucinations, reproducibility issues, and lack of localized recommendations.
Johnson et al [78]	2023	Evaluation of ChatGPT in answering clinical questions generated by clinician specialists.	A set of clinical questions generated by specialists.	ChatGPT generated answers for various medical difficulties.	Expert-established benchmarks and clinical standards.	Accuracy and completeness High accuracy for easy/moderate.	LLM (GPT-3.5–based chatbot trained until 2021).	Addressed risks of authoritative-looking errors and ethical/privacy concerns in AI^b^ medical tools.
Goh et al [79]	2023	Evaluation of ChatGPT (GPT-4) in clinical decision-making for chest pain cases.	Fifty clinicians were randomized to 2 different video clinical vignettes.	GPT-4 responses reviewed after initial physician answers; open interactions allowed.	Pre-LLM vs post-LLM responses were evaluated against clinical guidelines.	Accuracy and bias. Improvement (18%) in decision accuracy without increasing race/gender bias.	LLM (GPT-4) for recommendations and guideline discussions.	Discussed hallucinations, transparency, and the need for health care–specific interfaces.
Hanna et al [80]	2023	Comparison of Bing AI’s modes (Creative, Balanced, and Precise) for surgical nephrolithiasis questions.	Set of 20 questions on AUA^c^ surgical stone management.	Three Bing AI modes: Creative, Balanced, and Precise. Responses were evaluated according to AUA guidelines.	Evaluation using the Brief DISCERN score.	Quality, empathy, and adherence to guidelines. Creative mode showed the highest appropriateness (85%).	LLM (Bing AI with Creative, Balanced, and Precise modes).	Noted 15% inappropriate response rate; emphasized need for caution and further studies.
Zakka et al [81]	2024	Evaluation of retrieval-augmented language models (Almanac); Almanac vs other LLMs.	Clinical questions included in ClinicalQA^d^ (a benchmark of open-ended clinical questions).	Almanac used retrieval-based information for the accuracy of clinical answers.	Compared with ChatGPT-4o, Bing, and Bard.	Accurate clinical answers. Almanac performed better in factuality (91%), completeness, and adversarial safety (100%).	Retrieval-augmented LLMs integrating databases such as PubMed, UpToDate, and BMJ Best Practice.	Mentioned hallucination risks and emphasized rigorous testing before clinical implementation.
Huo et al [82]	2024	Analysis of LLM-based chatbots (ChatGPT, Bing, Bard, and Claude 2) in colorectal cancer.	Set of 9 clinical scenarios of colorectal cancer and screening.	Chatbots provided recommendations for screening, both for clinicians and lay patients.	Guidelines from USPSTF, CCS, USMSTF, and ACS; comparison between chatbots.	Accuracy and consistency across chatbots; ChatGPT was most accurate.	LLMs, including ChatGPT, Bing, Bard, and Claude 2.	Highlighted data quality variability and noted inconsistencies in patient vs clinician guidance.

a LLM: large language model.

b AI: artificial intelligence.

c AUA: American Urological Association.

d Clinical QA: clinical question answering.

**Table 5.** Systematic reviews of chatbot assessment studies.

Author	Year	Topic	Studies included	Intervention	Comparison	Risk of bias tool	Chatbot type	Reporting
Geoghegan et al [83]	2021	Focuses on postintervention follow-up in adults and adolescents.	10 studies: (3 RCT^a^, 6 cohort studies), 5492 participants (range 9‐4737)	Chatbots are not trained in psychology, but are designed for symptom monitoring and providing support.	Phone calls, standard postoperative care.	Cochrane RoB-2^b^, ROBINS-I^c^, and NIH^d^ cohorts**.** Risks due to lack of blinding and heterogeneity.	Text- and voice-based; rule-based and mixed dialog; integrated with electronic medical records and mobile apps.	Used PRISMA^e^. Recommends standardizing outcomes and implementation strategies.
Oh et al [84]	2021	Focuses on weight loss and a healthy diet in adults and adolescents.	9 studies (4 RCT, 5 quasi-experimental), 891 participants (range 19-274)	Chatbots trained in social persuasion and emotional connection.	Usual care, alternative controls.	NIH for interventions and NIH prepost. Biases due to small sample sizes and lack of longitudinal analysis.	Constrained (rule-based) and unconstrained chatbots (free input); integrated with graphics, images, and voice for interaction.	PRISMA suggests robust theoretical evaluation and consistent metrics.
Ogilvie et al [85]	2022	Focuses on psychological support for people with substance use disorders.	6 studies (1 RCT, 5 qualitative or mixed), 3‐180 participants	Chatbots trained in psychology, designed for CBT^f^ and motivational interviewing.	No comparator and standard care in RCT.	MMAT^g^. Biases due to small sample sizes and lack of active controls.	Text-based (NLP^h^) and big data for analyzing consumption patterns; integrated into apps and social networks.	Use PRISMA. Identifies the need for rigorous validation and ethical design.
Aggarwal et al [86]	2023	Focuses on behavioral changes in smoking cessation, diet, and adherence in adults and adolescents.	15 studies (4 RCT, 9 pre-post), 108,360 participants (range 20‐99217)	Chatbots trained in behavioral strategies such as CBT and motivational interviewing.	Standard care and untreated groups.	NIH tool. Moderate to high biases due to unvalidated measures.	NLP- and ML-based^i^ chatbots integrated into apps, messaging platforms, and social robots.	Identifies lack of standardization in metrics and outcomes. Use PRISMA and CONSORT-AI^j^.
Webster et al [87]	2023	Focuses on genetic counseling for hereditary cancer in adults.	7 observational studies, >50,000 interactions	Chatbots are not trained in psychology, but are designed to collect family histories and provide education.	No comparator	JBI^k^ cross-sectional. Biases due to a lack of demographic description and confounders.	Text-based with NLP; integrated into apps, mobile tools, and electronic medical records.	Suggests improving study quality and new controlled studies; use PRISMA.
Bendotti et al [88]	2023	Focuses on smoking cessation in adult smokers.	5 RCT, 58,796 participants (84‐57214)	Chatbots trained in psychology and behavioral strategies (CBT).	Apps without chatbots, standard care.	Cochrane RoB-2. Risks due to missing data and methodological deviations.	Mixed chatbots: rule-based and NLP, integrated into apps, social networks, and digital platforms.	USE PRISMA. Proposes CONSORT-AI^j^ to improve consistency in reporting.
Singh et al [89]	2023	Focuses on behavioral changes in physical activity, diet, and sleep in adults and adolescents.	19 studies (11 RCT, 5 prepost, 2 nonrandomized), 3567 participants (25-958)	Chatbots trained in behavioral change theories.	Standard care and alternative groups.	EPHPP^l^: 14 weak studies, 4 moderate, and 1 strong.	Text, AI^m^, voice-based chatbots with graphics and avatars.	PRISMA recommends more rigorous designs and evaluations.
Noh et al [90]	2023	Focuses on weight management in adults with obesity and overweight.	8 studies (3 RCT, 5 prepost), 712 participants (23-220)	Chatbots trained in psychology and personalization (CBT and individual goal-setting).	Alternative tutorials.	Cochrane RoB-2 and CASP^n^ checklist. Bias in randomization and selective reporting.	Text-based (NLP and ML), one multimodal (text + voice), and big data for population-level adjustments.	Use PRISMA. Highlights the need for longer follow-ups and larger sample sizes.
Kim [91]	2024	Focuses on mental, reproductive, and eating disorder health in women.	10 (7 RCT, 3 prepost), 21,537 participants (15‐19,643)	Chatbots trained in psychology are used for education, prevention, and psychological skill-building.	Waitlists and standard care.	Cochrane ROB-2. Biases in design, sample size, and intervention deviations.	Text-based with NLP and ML; integrated into apps and clinical environments.	Suggests metric standardization and methodological rigor. PRISMA.

a RCT: randomized controlled trial.

b RoB-2: Cochrane Risk of Bias 2 tool.

c ROBINS-I: Risk Of Bias In Non-randomized Studies of Interventions.

d NIH: National Institutes of Health.

e PRISMA: Preferred Reporting Items for Systematic reviews and Meta-Analyses.

f CBT: cognitive behavioral therapy.

g MMAT: Mixed Methods Appraisal Tool.

h NLP: natural language processing.

i ML: machine learning.

j CONSORT-AI: Consolidated Standards of Reporting Trials extension for Artificial Intelligence.

k JBI: Joanna Briggs Institute.

l EPHPP: Effective Public Health Practice Project.

m AI: artificial intelligence.

n CASP: Critical Appraisal Skills Programme.

The data obtained and used in this scoping review have been submitted earlier [92].

### Tools for Critical Appraisal and Associated Constructs

Of the 46 identified tools (Table 2), 26 were guides for reporting AI studies, 16 were critical appraisal tools, 2 were tools for the assessment of study quality, and 2 were protocols for tools assessing risk of bias (refer to ). Most of these tools (44) focused on classical predictive AI. Only 2 were oriented toward chatbot assessment studies: one, Quality Analysis of Medical Artificial Intelligence (QAMAI [66]), was designed to assess the quality of AI chatbots, and the other was a protocol for reporting this type of study [67].

With respect to the type of publication, most of these tools (41) were original. In 5 cases, the published tool was associated with a systematic review—in some cases developed to create the tool [54], and in others related to the assessment of included studies [24,52]. In other cases, it was part of a classic review in a journal [61] or in a book chapter [53].

Regarding clinical setting or specialty, 26 tools were designed for general clinical purposes. Eleven tools were developed for medical imaging or radiology and focused on image quality or diagnosis. Dermatology and traumatology had 2 tools each, and cardiology, radiation oncology, urology, oral health, and otorhinolaryngology-head and neck surgery had one tool each.

**Figure 2.**
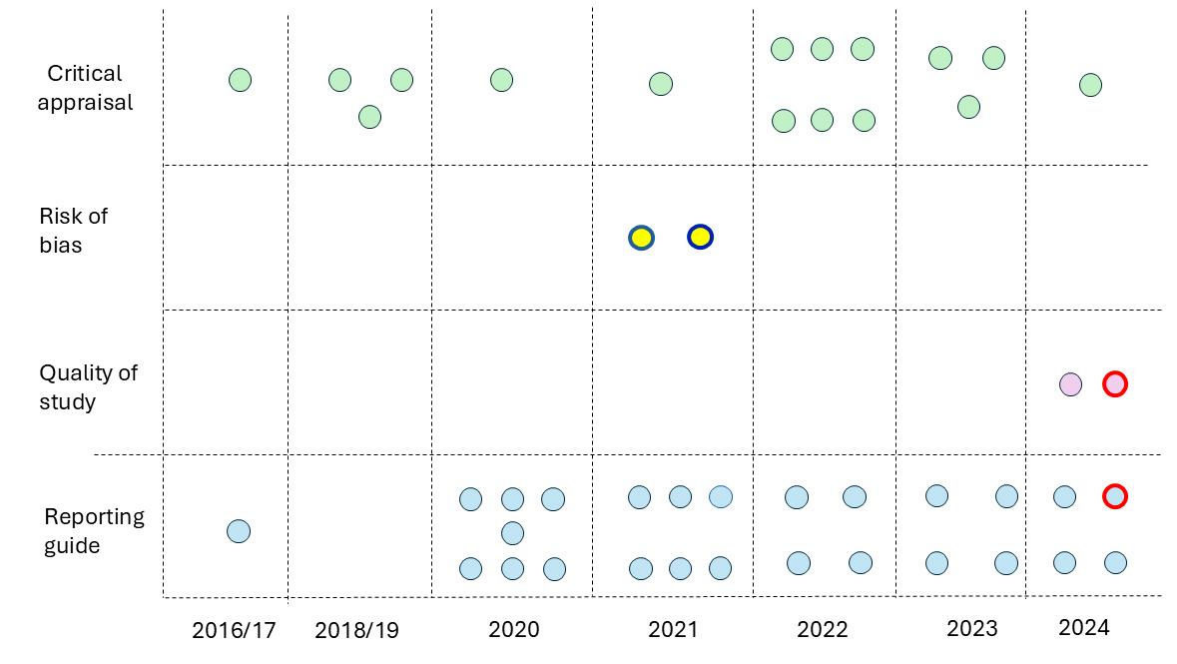
Constructs and year of publication as artificial intelligence (AI) tools.

Figures2  3 show the important historical aggregation of reporting and critical appraisal tools, particularly in 2021 and 2022, with a renewed increase in 2023 and 2024.

**Figure 3.**
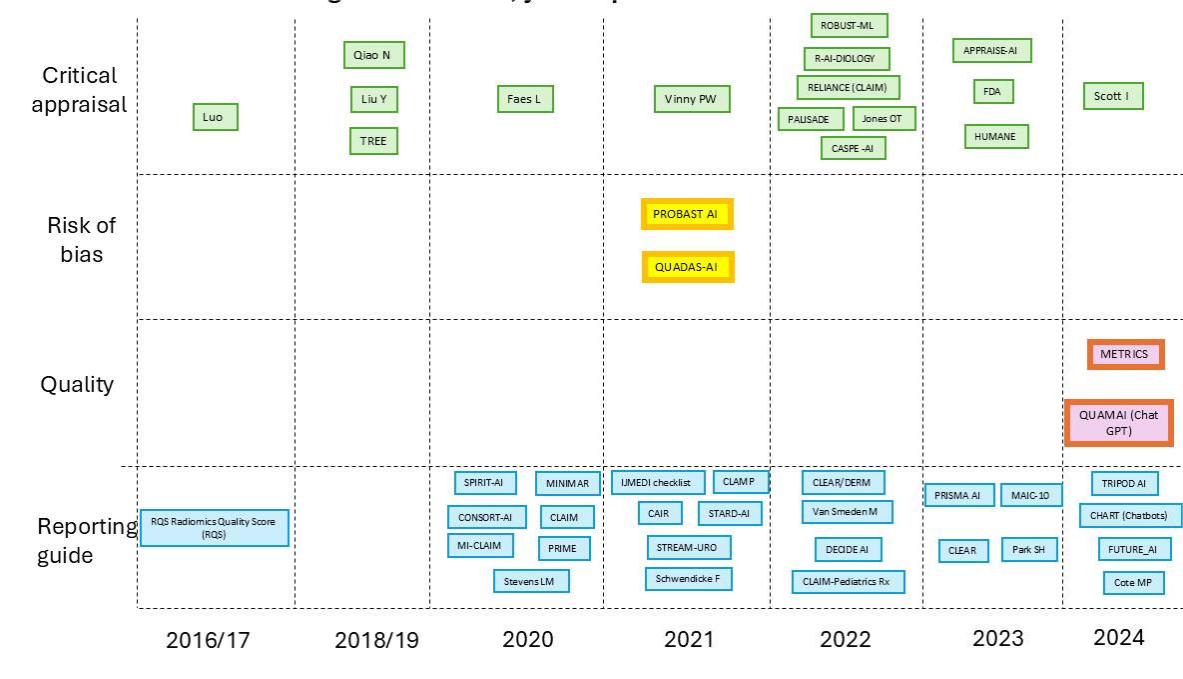
Constructs, year of publication, and name of tools.

Reporting tools are predominant, which is unsurprising, as they are a prerequisite to assess other dimensions and represent the first step to reach consistency. These reporting tools are for different designs: randomized controlled trial publications and protocols (CONSORT-AI, Consolidated Standards of Reporting Trials extension for Artificial Intelligence and SPIRIT, Standard Protocol Items: Recommendations for Interventional Trials), respectively, diagnostic accuracy studies (STARD-AI, Standards for Reporting of Diagnostic Accuracy Studies for AI), and Transparent Reporting of a multivariable prediction model for Individual Prognosis Or Diagnosis using AI (TRIPOD-AI). Other tools focused on medical images or other specialties (Checklist for Artificial Intelligence in Medical Imaging, CLAIM; Minimum Information for Medical AI Reporting, MINIMAR; Proposed Requirements for Cardiovascular Imaging Related MI Evaluation, PRIME; Standardized Reporting of ML Applications in Urology, STREAM-URO; Clinical Artificial Intelligence Research, CAIR, etc) and the PRISMA-AI protocol focused on the systematic review of AI studies (refer to acronyms in Table 6).

**Table 6.** Critical appraisal tools: name, acronym, and meaning or explanation.

Author, year	Name or acronym	Development, explanation, or meaning
Luo et al, 2016 [22]	Luo	Guidelines for developing and reporting machine learning (ML) predictive models.
Lambin et al, 2017 [23]	Radiomics Quality Score (RQS)	Radiomics quality score.
Qiao, 2019 [24]	Qiao	Checklist for studies of ML.
Liu et al, 2019 [25]	Liu	Checklist for studies of ML.
Vollmer et al, 2019 [26]	TREE	ML: 20 critical questions on transparency, replicability, ethics, and effectiveness.
Cruz Rivera et al, 2020 [27]	SPIRIT-AI	The Standard Protocol Items: Recommendations for Interventional Trials involving Artificial Intelligence.
Faes et al, 2020 [28]	Faes	Critical appraisal of ML studies.
Hernandez-Boussard et al, 2020 [29]	MINIMAR	MINimum Information for Medical AI Reporting: developing reporting standards for AI in health care.
Liu et al, 2020 [30]	CONSORT-AI	Consolidated Standards of Reporting Trials extension for AI.
Mongan et al, 2020 [31]	CLAIM	Checklist for AI in Medical Imaging.
Norgeot et al, 2020 [32]	MI-CLAIM	Minimum Information about Clinical AI Modeling: the MI-CLAIM checklist.
Sengupta et al, 2020 [33]	PRIME	Proposed Requirements for Cardiovascular Imaging-Related ML Evaluation.
Stevens et al, 2020 [34]	Stevens	Recommendations for reporting ML analyses in clinical research.
Cabitza and Campagner, 2021 [35]	IJMEDI checklist	International Journal of Medical Informatics checklist for studies of ML.
El Naqua et al 2021 [36]	CLAMP	Checklist for AI in medical physics.
Kwong et al, 2021 [37]	STREAM-URO	The Standardized Reporting of ML Applications in Urology framework.
Meshaka et al, 2021 [38]	CLAIM-Pediatrics^a^ Rx	AI research reporting guidelines relevant to the pediatric radiologist (CLAIM adaptation).
Olczak et al, 2021 [39]	CAIR	Clinical AI Research checklist.
Schwendicke et al, 2021 [40]	Schwendicke	AI in dental research: checklist.
Sounderajah et al, 2021 [41]	QUADAS-AI	Quality Assessment of Diagnostic Accuracy Studies for Artificial Intelligence.
Sounderajah et al, 2021 [42]	STARD-AI	Standards for Reporting of Diagnostic Accuracy Studies for AI.
Vinny et al, 2021 [43]	Vinny	Critical appraisal of ML.
Collins et al, 2021 [44]	PROBAST-AI	Prediction model Risk Of Bias Assessment Tool for AI studies.
Al-Zaiti et al, 2022 [45]	ROBUST-ML	Ruling Out Bias Using Standard Tools in ML.
Daneshjou et al, 2022 [46]	CLEAR/DERM	Checklist for Evaluation of Image-Based AI Algorithm Reports in Dermatology.
Haller et al, 2022 [47]	R-AI-DIOLOGY	Checklist for evaluation of AI tools in clinical neuroradiology.
Jha et al, 2022 [48]	RELAINCE	Recommendations for Evaluation of AI for Nuclear Medicine.
Padula et al, 2022 [49]	PALISADE	ML in Health Economics and Outcomes Research: Purpose, Appropriateness, Limitations, Implementation, Sensitivity, Algorithm characteristics, Data characteristics, and Explainability.
Van Smeden et al, 2022 [50]	Van-Smeden	Critical appraisal of AI-based prediction models for cardiovascular disease.
Vasey et al, 2022 [51]	DECIDE-AI	Reporting guideline for early-stage clinical evaluation of decision support systems driven by AI.
Jones et al, 2022 [52]	Jones	Checklist for evaluation of AI and ML for triage or detection of possible skin cancers.
Cabello, 2022 [53]	CASPE-AI	Critical Appraisal of Studies using Predictive Evidence-AI.
Cacciamani et al, 2023 [54]	PRISMA-AI	Preferred Reporting Items for Systematic reviews and Meta-Analyses extension for AI.
Alberich et al 2023 [55]	MAIC-10	Must AI Criteria‐10: quality checklist for publications using AI and medical images.
Kocak et al, 2023 [56]	CLEAR	CheckList for Evaluation of Radiomics research.
Kwong et al, 2023 [57]	APPRAISE-AI	Tool for quantitative evaluation of AI studies for clinical decision support.
Park et al, 2023 [58]	Park	Critical appraisal: 10 key items for radiologists to check when reading publications of clinical research on AI.
FDA, 2023 [59]	FDA	Ten guiding principles for developing good ML practices.
Collins GS, 2024 [60]	TRIPOD-AI	Transparent Reporting of a Multivariable Prediction Model for Individual Prognosis Or Diagnosis using regression or ML methods.
Du Toit et al, 2023 [61]	HUMANE	ML Analytics Network survey questionnaire for hypertension studies
Cote and Lubowitz, 2024 [62]	Cote	Recommended requirements and essential elements for proper reporting of the use of AI and ML tools.
Kocak et al, 2024 [63]	METRICS	METhodological RadiomICs Score.
Lekadir et al, 2024 [64]	FUTURE-AI	Guiding principles and consensus recommendations for trustworthy AI.
Scott et al, 2024 [65]	Scott	Checklist for assessing suitability of ML applications.
Vaira et al, 2024 [66]	QUAMAI (ChatGPT)	Validation of the Quality Analysis of Medical AI (QAMAI) tool.
CHART Collabor, 2024 [67]	CHART (Chatbots)	Protocol for the development of the Chatbot Assessment Reporting Tool (CHART) for clinical advice.

a CLAIM: Checklist for Artificial Intelligence in Medical Imaging.

The number of tools for critical appraisal has significantly increased since 2022. They include aspects of the relevance of clinical questions and the clinical context of the technology, but also the necessary reflections about how to apply the results to the clinical setting or clinical decisions. Most of these papers have a teaching purpose (explicit or implicit) and often include comprehensive reviews (systematic or classical) of AI techniques with glossaries or taxonomic suggestions.

Only two tools are focused on the risk of bias. Both focus on predictive AI (Quality Assessment of Diagnostic Test Accuracy Studies for Artificial Intelligence, QUADAS-AI) and Prediction Model Risk Of Bias Assessment Tool for AI studies, (PROBAST-AI), and both are AI extensions of classical tools for diagnosis accuracy studies and prognosis studies, respectively, and, at the time of writing, are not yet available (they are under construction or exist as protocols). Regarding the quality study, we found 2 instruments: Methodological Radiomics Score (METRICS), which is a recent tool designed to assess the quality of radiomic studies, and the above-mentioned QAMAI, which aims to assess the quality of health information offered by AI chatbots trained in otorhinolaryngology. This latter is inspired by mDISCERN, which is a well-validated and widely used tool for assessing the quality of health information from websites [93].

**Figure 4.**
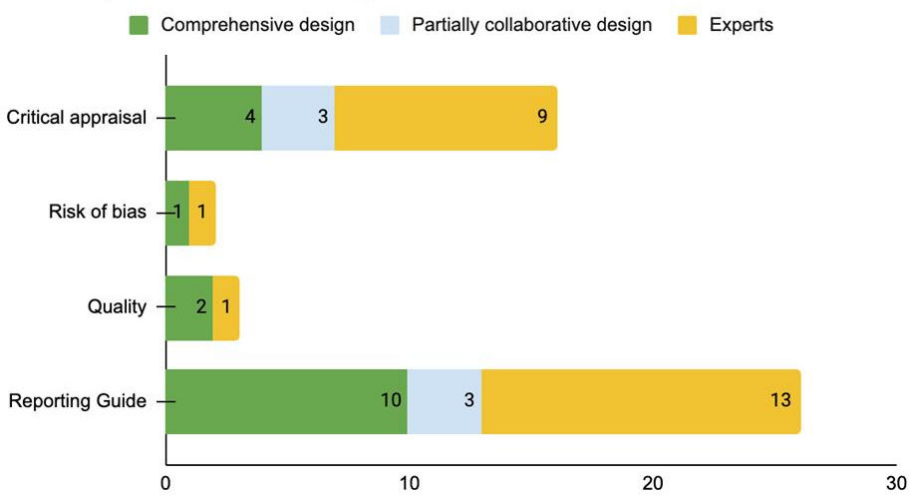
Design style for each type of constructs.

With respect to the methods used to develop the tools (Figure 4), a comprehensive strategy including a systematic review of the literature and a formal Delphi process was used in 15 cases. In 6 tools, the method was only partially described, and in 23, there was no sufficient description of the methods used, so we assume that they were developed by experts. There were, on average, 21 items for critical appraisal tools, 30 for study quality tools, and 29 for reporting guides. There was no information about the above-mentioned protocols for risk of bias.

### Bias and Bias Mitigation

Nine papers addressed the issue of bias or bias mitigation in AI studies. Three of them focused specifically on bias classification: in 2 cases in an exhaustive manner [68,69] and in another case from a more general view [73].

Three of the obtained papers are oriented to bias and mitigation from a specific clinical or technological perspective: in 1 case only for ML [75], and in the 2 remaining cases from a nephrology [74] or public health perspective [76].

We also found 3 very relevant papers: one [70] is a comprehensive classic review focused on bias classification and explanation, including how to design strategies for bias mitigation. Another [71] is an official publication from the US National Institute of Standards and Technology, which addresses the definition of standards in bias taxonomy and classification of their categories and suggests a guide to management and mitigation of bias in the AI context. The last one [72] is a systematic review of computational bias, with a precise description of fairness metrics and a synthesis of strategies for bias mitigation. In addition, the review provides a catalog of software tools and libraries for helping developers and users to explore the issue of fairness and bias in AI.

In addition, many of the retrieved papers classified in our scoping review as critical appraisal tools also included bias classification, bias mitigation, or glossaries of clinical AI terms [24,25,43,45,47,52].

### Chatbot Assessment Studies

We identified 15 studies related to chatbot assessment studies. Six of them are primary research studies of chatbot assessments (Table 4), and 9 are systematic reviews in which chatbots are compared with other interventions (Table 5). In 5 of the primary studies, a nonclinical study population consisting of sets of questions, scenarios, vignettes, or a bank of standard questions was used [77,78,80-82]. In the other primary study [79], the study population was a group of clinicians randomly allocated to view 1 of 2 videos with clinical scenarios, and the clinicians’ answers were evaluated before and after a chatbot interaction (ChatGPT). With respect to the study design, in 3 cases, the objective was to assess chatbot performance [77,78,80], in 2 cases, the aim was to compare performance between different chatbots [81,82], and in the above-mentioned study involving clinicians, the aim was the exploration of changes in clinical answers after chatbot interaction in a before-and-after scheme [79]. All 6 studies assessed modern chatbots (generative chatbots), and all of them mentioned challenges in this area in the discussion (eg, inconsistency in answers, low transparency, “hallucinations” [when AI models produce incorrect or misleading results], and rates of inappropriate responses). All agreed on the need for specific health care–trained interfaces.

We found 9 systematic reviews (Table 5). Two were published in 2021, so the chatbots used were not LLM chatbots (ie, older chatbots) [83,84], and the other 7 included modern LLM (generative chatbots) with several study designs: Two included only randomized controlled trials [88,91], 5 included randomized or quasi-experimental studies, or both [83,84,86,89,90], and 1 included qualitative/mixed-methods studies [85]. The last one was a systematic review of observational studies focused on counseling for hereditary cancer in selected at-risk adults [87]. The tools used to assess study quality depended on the designs included in the systematic review, so different classic tools were used: Cochrane Risk of Bias 2 (Cochrane Collaboration) in 4 cases [83,87,90,91], NIH tools for experimental and observational designs in 2 cases [86,94], JBI–cross-sectional tool for observational studies [87], and other tools for other pre-post and qualitative designs.

The questions for these reviews are provided in Table 5. Most concerned counseling is associated with treatment; two were oriented toward weight loss management, different addictions, and reproductive health counseling. In 5 cases, the intervention was a chatbot trained in psychology (sometimes cognitive behavioral therapy) compared with standard care. Finally, regarding the study report, most of the included trials used the CONSORT classic guideline, although CONSORT-AI was published in 2020. However, CONSORT-AI was mentioned in two systematic reviews [86,88]. For systematic review reporting, all included studies used PRISMA classic, which is reasonable because PRISMA-AI was published in 2023.

## Discussion

### Principal Findings

We conducted a comprehensive scoping review and identified 70 papers corresponding to the 3 proposed questions: tools for critical appraisal, bias and bias mitigation, and chatbot assessment studies. Although critical appraisal tools were the main objective of this review, AI types of bias were also included because validity (or absence of bias) is an important component of critical appraisal. Chatbot studies were included because they represent an important, recent, and disruptive technology. The three areas together map the current landscape of evidence in the critical appraisal of clinical AI studies.

We selected critical appraisal as the main domain for this review because it is a wider and more inclusive concept than risk of bias, quality, or reporting, and it is more related to clinical practice. This decision implied a change in the published protocol and was adopted after discussion.

Reporting guides are essential for authors in writing their studies and for editors in maintaining consistency across publications. In fact, they are a prerequisite for adequate reading. Critical appraisal tools are more focused on making judgments about the validity and applicability of the evidence, and they usually have a diffusion or teaching purpose. A paper can be perfectly reported; it may even be valid, yet be of no use in a clinical setting. Finally, risk of bias and quality are very precise concepts, and their tools are complex and designed as far as possible to avoid inconsistencies, so they are more suitable tools for research syntheses. Nevertheless, reporting, critical appraisal, risk of bias, and quality form a cluster of closely related constructs with overlapping areas.

### Comparison With Previous Work

Adequate reporting varies according to the structure or the type of study we are addressing, and is not only an editorial requirement but also part of study quality. Obviously, good reporting is a precondition to assess study quality, but there is also empirical evidence that some reporting flaws (or nonreporting) are associated with bias in the effect estimation [94,95]. Therefore, exploring the reporting is essential to judge the validity of any study, as it facilitates study replication, risk of bias or quality assessments, interpretation of the results, and judgment of the value and applicability of the results in real clinical settings for individualized or collective decisions. It is also necessary to include and assess studies in systematic reviews and to evaluate the systematic review itself. Thus, it is part of the critical appraisal process [9,96,97].

This overlap between reporting and critical appraisal has been a source of inconsistencies between raters when classifying papers in this scoping review. Iterative discussions were necessary to reach an agreement. The most important criterion we used to classify the papers within the critical appraisal category was the relevance of the question in the clinical context and a clear intent to help in applicability.

Quality of study and risk of bias have been used interchangeably, but quality is a descriptive approach to methodological characteristics that may have a possible influence on the effect estimation (called safeguards), whereas risk of bias is an empirical judgment (guided by methodological signaling and criteria) about a possible bias in a particular effect estimation. This new construct of risk of bias is expressed as low, high, or moderate. Currently, the risk of bias is more commonly used than quality [98].

### Strengths and Limitations

With respect to evidence search, the strategy and the sources are sensitive enough to identify the existing tools for critical appraisal and related constructs. Only 10 papers escaped our formal search strategy and were retrieved by other methods. In addition, we carried out a special effort to search for AI tools in systematic reviews of AI studies during the full-text screening phase. Therefore, we believe that this study is sensitive in capturing the evidence about AI critical appraisal tools.

As for the selection of sources of evidence for AI tools and data charting (with their implicit value judgment in classifying tools), the iterative process of consensus is a consistent strategy.

For bias in AI and bias mitigation, the search strategy was able to identify the main papers about bias classification and mitigation, although it was not specifically designed for this purpose. We are aware of the enormous number of existing publications on each specific bias. However, the retrieved papers give us an adequate representation of them, which will allow us to make forward and backward “snowballing” to collect the relevant evidence for future concept analysis studies.

Clearly, chatbot assessment studies constitute a special group in this review, which is full of difficulties. First, they are not a MeSH term yet, so controlled language cannot be used. We have used a reasonable strategy based on free text, synonyms, and truncations, but it may be improved in future updates with the appropriate MeSH term and by using the search strategy of new systematic reviews about chatbots and the use of semantic search technologies. To balance this, we decided to have flexible inclusion criteria. Second, chatbot assessment studies are heterogeneous and inconsistent in the design, analysis, and reporting, so we used ChatGPT-4o for data extraction; however, all outputs were independently reviewed by 2 authors against the original papers, and no major corrections to the extracted information were required. Third, the study populations are variable and are based on preclinical scenarios, vignettes, or a set of questions that lie at the frontier of real clinical practice. On the other hand, the aggregation of systematic reviews on chatbots is not exhaustive but may be considered a detailed and up-to-date list of this type of study, their main characteristics, and the tools used for reporting the individual studies and the reviews, and for assessing the risk of bias. In this sense, a recent review shows results consistent with our study [99].

There are some limitations to this scoping review. The search strategy followed a general approach for all the questions of the study, but was primarily guided by the main question and was not specifically designed for bias and bias mitigation or chatbot assessment. On the other hand, the absence of MeSH for chatbot studies and the heterogeneity of objectives, questions, designs, devices, and analyses make it very difficult to search for this type of study. In addition, the methods used to organize the data extraction have a potential limitation due to the novelty of applying LLMs in evidence synthesis, as formal standards for their integration are still under development. Finally, this field is evolving very quickly, so many of the conclusions about the existing evidence have a limited period of validity.

### Implications for Research

There is a vast array of tools available, with 2 clear aggregations in the areas of reporting guides and critical appraisal tools. Thus, the newly arising question is: What is the best tool for a particular setting or specific purpose? At the same time, there are some gaps in knowledge identified in this scoping review. These aggregations and the existing gaps have implications for research and for clinical practice.

Reporting guides have been recently synthesized in a systematic review [100] that also includes tools for basic and laboratory research, and whose search ended in 2022. This topic should be harmonized, and the review should probably be either updated or reformulated from a clinical standpoint.

Similarly, critical appraisal tools are enormously varied and full of different nuances and approaches, so selecting one of them can be very challenging. We believe that the topic deserves a qualitative synthesis to clarify the key elements for choosing.

New risk of bias tools for AI in prognosis and diagnosis (QUADAS-AI and PROBAST-AI), as well as a PRISMA-AI extension for systematic reviews, are expected, as well as the Chatbot Assessment Reporting Tool (CHART), for reporting chatbot assessment studies. The AI extension of other classic tools, such as Cochrane Risk of Bias and ROBINS, among others, should be considered.

Finally, the development of standards for the design, reporting, and assessment of chatbot assessment studies and chatbot health advisory studies is a clear gap in our toolbox and needs to be addressed.

### Implications for Clinical Practice

In the realm of clinical practice, it is important to clarify the appropriate selection of adequate tools for critical appraisal, and it is essential to develop teaching strategies for the dissemination of skills for the critical appraisal of AI studies, including knowledge about the types of bias to be tackled [101].

### Conclusion

“We can only see a small distance ahead, but we can see plenty that needs to be done” [102].

## Supplementary material

10.2196/77110 Multimedia Appendix 1 Data extraction template.

10.2196/77110 Multimedia Appendix 2 GPT–Retrieval Augmented Generation (RAG; prompting) template.

10.2196/77110 Multimedia Appendix 3 Exclusions after full-text screening.

10.2196/77110 Multimedia Appendix 4 Footnotes for figures and tables.

10.2196/77110 Checklist 1 PRISMA (Preferred Reporting Items for Systematic reviews and Meta-Analyses) checklist search tools.

10.2196/77110 Checklist 2 PRISMA-SCR (Preferred Reporting Items for Systematic reviews and Meta-Analyses extension for Scoping Reviews) checklist.

## Abbreviations

AI: artificial intelligence
CAIR: Clinical Artificial Intelligence Research
CHART: Chatbot Assessment Reporting Tool
CLAIM: Checklist for Artificial Intelligence in Medical Imaging
CONSORT-AI: Consolidated Standards of Reporting Trials extension for Artificial Intelligence
JBI: Joanna Briggs Institute
LLM: large language model
MeSH: Medical Subject Headings
METRICS: METhodological RadiomICs Score
MINIMAR: MINimum Information for Medical AI Reporting
ML: machine learning
PRIME: Proposed Requirements for Cardiovascular Imaging Related Machine Learning Evaluation
PRISMA: Preferred Reporting Items for Systematic reviews and Meta-Analyses
PRISMA-ScR: Preferred Reporting Items for Systematic reviews and Meta-Analyses extension for Scoping Reviews
PROBAST-AI: Prediction model Risk Of Bias ASsessment Tool for AI studies
QAMAI: Quality Analysis of Medical Artificial Intelligence
QUADAS-AI: Quality Assessment of Diagnostic Accuracy Studies for Artificial Intelligence
SPIRIT-AI: Standard Protocol Items: Recommendations for Interventional Trials involving Artificial Intelligence
STREAM-URO: Standardized Reporting of ML Applications in Urology
TRIPOD-AI: Transparent Reporting of a multivariable prediction model for Individual Prognosis Or Diagnosis using AI
