# Supplementary material for: Critical Appraisal Tools for Evaluating Artificial Intelligence in Clinical Studies: Scoping Review
Source: J Med Internet Res. 2025 Dec 8;27:e77110. doi: 10.2196/77110 (PMC12685289; doi:10.2196/77110)
Supplement: Multimedia Appendix 1 [file jmir-v27-e77110-s001.docx]

**CRITICAL APPRAISAL TOOL FOR ARTIFICIAL INTELLIGENCE CLINICAL STUDIES. A SCOPING REVIEW.**

1. **DATA EXTRACTION TEMPLATE.**

This document aims to explicitly define the items to be extracted in the Review. These are already tabulated in XLS format in a spreadsheet installed in shared mode on **Google Drive.** It has been discussed by Vicente Ruiz and Juan Cabello and has been tested in a group of articles and modified after a consensus session. It will be uploaded to the OIS website to be shared.

Data extraction and evaluation were carried out by Miguel Torralba and Vicente Ruiz. Juan Cabello was a referee when consensus was not reached.

The template was inspired by: Pollock D, Peters MDJ, Khalil H, McInerney P, Alexander L, Tricco AC, Evans C, de Moraes ÉB, Godfrey CM, Pieper D, Saran A, Stern C, Munn Z. ***Recommendations for the extraction, analysis, and presentation of results in scoping reviews***. JBI Evid Synth. 2023 Mar 1 ;21 (3):520-532. doi: 10.11124/JBIES-22-00123. PMID: 36081365.

1. **DATA CHARTING for TOOLS GENERAL ITEMS:**
2. **Author** (article format)
3. **Year** of publication. The general table of instruments is indexed by year of publication.
4. **DOI (**Digital object identifier**)**
5. **ARTICLE TITLE**
6. **NAME OR ACRONYM OF THE GUIDE OR INSTRUMENT.** If not available, the name of the first author or the Institution where it was developed was assigned.
7. **ARTICLE TYPE** (considered in *editorial terms)*:
   1. Original. Typical article with a classic format that includes the sections Introduction, methods, results and discussion.
   2. Classical/narrative review. This review allows for flexibility in questioning, searching, extraction, and synthesis, without adhering to a strict format and without assessing or quantifying the evidence, when possible.
   3. Systematic Review. Consistent with the question, search, data extraction and synthesis according to EQUATOR
   4. Other Conceptual analysis or other mixed types.
   5. (editorials and abstracts were excluded from the final results)
8. **CLINICAL AIM:** *refers to whether it is a general approach to critical reading, reporting, etc. , or refers to a specific type of design used for clinical questions* ):
   1. Generic Critical Reading, Machine Learning (ML), or AI (Artificial Intelligence)
   2. LC specific design:
      1. Diagnostic Studies
      2. Prognostic or predictive clinical studies
      3. Randomized clinical trials/Treatment
      4. Real world study
   3. Others
   4. Protocols (except for exceptions)
   5. Mixed or other (describe).
9. **Clinical area or specialty of the instrument (in which was published):**
   1. For broad clinical/research use
   2. For use in specific specialties (cardiology, neurology, pediatrics, imaging, urology, etc.)
10. **PURPOSE: (**objective *to which the instrument or article is directed)*.
    1. Critical reading. It is an instrument designed to help judge the Quality in the sense of methodological correctness of the design and its consequences in terms of risk of bias in the estimates. It also includes the relevance of the question and its construction, as well as the applicability of the results to diverse areas.
    2. Study quality. It is a set of criteria designed to judge a study as a whole (including correct design, correctness of reporting, validity, ethics). etc).
    3. Reporting Guide. It is a set of criteria and recommendations designed to standardize the presentation of research findings methodologies
    4. Risk of bias. Instruments for estimating the certainty of study estimates, based on their methodological characteristics.
    5. Description of biases in general in AI or clinical epidemiology.
    6. Assessment of Chatbot Studies. Instruments were included in the main table, but chatbot studies were described separately in two more tables in a disaggregated manner.
    7. Others. Not included in the above categories
11. **TYPE OF INSTRUMENT/S.**
    1. Check full list (includes domains and items)
    2. Check incomplete list (includes only items)
    3. List of recommendations.
    4. List of reflections or categories of biases
    5. Others (describe)
12. **Number of ITEMS in the tool**
13. **NUMBER OF DOMAINS or Sections in the tool**
14. **SETTING or CONTEXT OF USE**
    1. Preclinical (e.g. how to report an ML model)
    2. Social (identifying algorithmic biases or mitigating algorithmic biases)
    3. Nuclear clinician (focused on diagnosis, prognosis, treatment or decision model)
    4. Preclinical and clinical use.
    5. Others (describe)
15. **SOURCE OF INFORMATION/VALUATION OF THE INSTRUMENT**
    1. Patient databases
    2. Patients or Patient Records
    3. Multiple sources
    4. Vignettes or clinical scenarios
    5. Publications (e.g. NEJM cases)
    6. not specified
    7. Not applicable
16. **CONSTRUCT explored or measured:**
    1. Critical reading (includes relevance of question and applicability)
    2. Quality
    3. Report
    4. Risk of bias
    5. Algorithmic validity
    6. Others
17. **DEVELOPMENT OF THE INSTRUMENT: streategy or design for developing the instrument**
    1. Complete (“comprehensive”): with a Systematic review + a formal Delphi-type process, and with an explicit protocol, item generation, item selection and a complete description of the process.
    2. Partially Collaborative: with protocol only partially explicit.
    3. Not stated or Expert-based: Assumes no specific design and is based only on expert opinions with no clear development protocol.
18. **FINANCING:** Described as YES or NO if there is an explicit description and as Not described (NA when no explicit reference was done)
19. **CONFLICT OF INTEREST:** It is described as YES or NO if there is an explicit description and as Not described (NA when no explicit reference was done).
20. **DATA CHARTING for BIAS & MITIGATION**
21. Autor
22. Year
23. Title
24. Bias classification
25. Bias mitigation
26. Comments
27. **DATA CHARTING for CHATBOT ASSESSMENT STUDIES**

(They were described in two tables. The first table included the same items as in the previous table (Author, Year, etc.) And in both athe data were charted according to the following definitions or criteria. In parallel data charting of these studies have been done through a RAG system of prompts + chat GPT.

**Title of study:**

1. **Type of study:**
   1. Original. Typical article with a classic format that includes the sections Introduction, methods, results and discussion.
   2. Systematic or classic review. See previous definitions in 6.2 and 6.3
2. **Originals.** Research question.
   1. CHATBOT /S in study:
      1. Population. There is a clear description of the population to which it is directed (clinicians, patients, general population or those with a particular disease or problem).
      2. Intervention (or test). The intervention or characteristics of the chat bot are clearly described.
      3. Comparison (or reference). The comparison or reference to which the comparison is being made is clearly described (clinical judgment, clinical guideline, etc.).
      4. The percentage of agreement between the chatbot's recommendations and the gold standard is explicitly stated.
      5. Formal study design (from an epidemiological perspective).
      6. Type of analysis performed: Percentage before and after, percentage according to the gold standard,
      7. Conclusions:
      8. Other comments: utility, costs, satisfaction, costs, etc.
3. **Systematic review**. Research question
   - 1. Topic of the study.
     2. Population. There is a clear description of the population to which it is directed (clinicians, patients, general population or those with a particular disease or problem)
     3. Intervention (or test). The intervention or characteristics of the chat bot are clearly described.
     4. Comparison (or reference). The comparison or reference to which the comparison is being made is clearly described (clinical judgment, clinical guideline, etc.).
     5. Results. The percentage of agreement between what the chatboot help suggests as valid and what the Gold Standard suggests is clearly described .
     6. Formal study design (from an epidemiological perspective).
     7. Type of chatbot used
     8. Type of analysis performed: Percentage before and after, percentage according to the Gold standard, comparison with some statistic of average successes, etc.)
     9. Conclusions:
     10. Other comments: utility, costs, satisfaction, costs, etc.
   1. Type of designs included
      1. Clinical trials
      2. Observational studies
      3. NA (not applicable)
   2. Assessment of the quality of the studies (including reporting quality). Description of whether any type of quality assessment instrument was used (eg, National Institutes of Health (NIH) Quality Assessment Tool , BA Met . Or others)
   3. Review synthesis methods:
      1. qualitative
      2. quantitative
      3. mixed
      4. Describe analysis and conclusions
   4. Other comments: open field

**============================================**

**CHAT GPT. RAG (prompt)**

**Datacharting Originales (primary studies)**
GPT creado para extraer información de articulos originales sobre chatbot en formato pdf. Se subirá un articulo en pdf, el GPT lo leerá y devolverá la información a continuación solicitada en formato tabla. Ten en consideración los siguientes puntos

-vas a tener que leer el artículo despacio. A veces la información es confusa. Si te plantea dudas leelo dos o tres veces. Puedes preguntarte a tí mismo de nuevo para corroborar.

- A veces la información va a venir en tablas, por lo que tendrás que leer tablas. Puede que hacer sumas para calcular número de pacientes.

- No alucines. Trabaja solo con la información del PDF que se te suban en cada momento.

- devuelve solo la información del pdf que se te haya subido. No encadenes información con otros pdfs previos.

Autor: Apellido e inicial del primer autor

Año: año de publicación

Título: título del paper

Descripción: descripción breve del objetivo del estudio.

Población: tipo de población que recoge la revisión sistemática, si hay varios tipos, recoge los varios tipos. La población a estudio puede ser un dataset creado ad hoc, una batería de preguntas sobre un tema especifico ad hoc, especifica todos los datos que encuentres sobre ello

Diseño de estudios: tipo de diseños, pueden ser diseño tipo estudio diagnostico like en el que se esta evaluando la performance de un instrumento frente a gold standard

Intervención: tipo de intervención o intervenciones que se analizan. Puede ser accuracy de respuestas contra guías clínicas

Comparador: cual es el gold standard con el que se comparan

Desenlace: objetivo del estudio

Análisis: tipo de análisis que se realiza.

Tipo Chatbots: que tecnología tenían los chatbots empleados. chatbot conversacional, chatbot basado en NLP, chatbot basado en LLM? en sentido intenta ser preciso y técnico

Reporting: ¿han empleado algún formato de reporting specifico para el reporte? ¿Mencionan algo sobre alucinaciones?

**Datacharting revisiones sitemáticas**

GPT creado para extraer información de revisiones sistemáticas y metaanálisis en formato pdf. Se subirá un artículo en pdf, el GPT lo leerá y devolverá la información a continuación solicitada en formato tabla. Ten en consideración los siguientes puntos

-vas a tener que leer el artículo despacio. A veces la información es confusa. Si te plantea dudas leelo dos o tres veces. Puedes preguntarte a tí mismo de nuevo para corroborar.

- A veces la información va a venir en tablas, por lo que tendrás que leer tablas. Puede que hacer sumas para calcular número de pacientes.

- No alucines. Trabaja solo con la información del PDF que se te suban en cada momento.

- devuelve solo la información del pdf que se te haya subido. No encadenes información con otros pdfs previos.

Autor: Apellido e inicial del primer autor

Año: año de publicación

Título: título del paper

Descripción: descripción breve del objetivo del estudio. Recoge si describen pregunta PICO y si reportan empleo de PRISMA o registran la revisión en PROSPERO

Población: tipo de población que recoge la revisión sistemática, si hay varios tipos, recoge los varios tipos.

numero de estudios: número de estudios que se analizan después de todo el proceso de selección

Tamaño muestral: número total de pacientes o participantes derivado de los estudios que finalmente se han seleccionado para la revisión. (da el rango de número de pacientes según los estudios seleccionados)

Diseño de estudios: tipo de diseños que tienen los estudios que se han seleccionado para la revisión ej: ensayos clínicos, cohortes, antes y después.

Intervención: tipo de intervención o intervenciones que se analizan

Comparador: con qué se comparan en general las intervenciones, los comparadores pueden ser varios, en algunos no hacer nada, en algún standard of care.

Tipo de desenlace: cuantitativo, cualitativo o mixto. Señala si realiza metaanálisis

Desenlace: objetivos que analizala revisión sistemática. Si realiza metaanalisis, resultado

Risk of bias: emplea algún instrumento de risk of bias? si lo utiliza señala cual con el nombre exacto. Comenta el risk of bias encontrado de la manera más objetiva posible.

Consideraciones éticas: ¿hace consideraciones éticas específicas sobre inteligencia artificial o chatbots?

Idioma: en qué idioma o idiomas se han aceptado los artículos revisados para la revisión

Tipo Chatbots: que tecnología tenían los chatbots empleados. chatbot conversacional, chatbot basado en NLP, chatbot basado en LLM? en sentido intenta ser preciso y tecnico

Reporting: consideraciones especiales en el reporte de los resultados que sea específico de chatbot?
