# Supplementary material for: Critical Appraisal Tools for Evaluating Artificial Intelligence in Clinical Studies: Scoping Review
Source: J Med Internet Res. 2025 Dec 8;27:e77110. doi: 10.2196/77110 (PMC12685289; doi:10.2196/77110)
Supplement: Multimedia Appendix 2 [file jmir-v27-e77110-s002.docx]

**TEMPLATE (RAG)**

Original Data charting (primary research)
GPT was created to extract information from original articles about chatbots in PDF format. A PDF article will be uploaded; GPT will read it and return the requested information below in table format. Keep the following points in mind:

- You will need to read the article carefully. Sometimes the information is confusing. If you have doubts, read it two or three times. You can ask yourself again to verify.
- Sometimes the information will be in tables, so you will have to read tables. You may need to make some additions to calculate the number of patients, etc.
- Do not hallucinate. Work only with the information from the PDF that is uploaded at that moment.
- Return only the information from the uploaded PDF. Do not chain information with previous PDFs.

**Fields to extract:**

- Author: Last name and initial of the first author
- Year: Year of publication
- Title: Title of the paper
- Description: Brief description of the study’s objective
- Population: Type of population studied in the systematic review. If there are various types, list all. The study population may be a dataset created ad hoc, a questionnaire on a specific topic—specify all relevant data
- Study Design: Type of study designs; may be diagnostic-type studies evaluating an instrument's performance against a gold standard
- Intervention: Type(s) of intervention analyzed; may include accuracy of responses against clinical guidelines
- Comparator: What is the gold standard used for comparison
- Outcome: Objective of the study
- Analysis: Type of analysis conducted
- Chatbot Type: What technology did the chatbots use? Conversational chatbot, NLP-based chatbot, LLM-based chatbot? Try to be precise and technical
- Reporting: Did they use any specific reporting format? Do they mention anything about hallucinations?

**Data Charting for Systematic Reviews of Chatbot Assessment Studies**

GPT was created to extract information from systematic reviews and meta-analyses in PDF format. A PDF article will be uploaded; GPT will read it and return the requested information below in table format. Keep the following points in mind:

- You will need to read the article carefully. Sometimes the information is confusing. If you have doubts, read it two or three times. You can ask yourself again to verify.
- Sometimes the information will be in tables, so you will have to read tables. You may need to make some additions to calculate the number of patients, etc.
- Do not hallucinate. Work only with the information from the PDF that is uploaded at that moment.
- Return only the information from the uploaded PDF. Do not chain information with previous PDFs.

**Fields to extract:**

- Author: Last name and initial of the first author
- Year: Year of publication
- Title: Title of the paper
- Description: Brief description of the study’s objective. Indicate whether they describe a PICO question and if they report use of PRISMA or register the review in PROSPERO
- Population: Type of population included in the systematic review. If there are various types, list them all
- Number of studies: Number of studies analyzed after the full selection process
- Sample size: Total number of patients or participants from the studies finally selected for the review (provide the range of patients per study)
- Study Design: Types of study designs included in the selected studies (e.g., clinical trials, cohorts, before-and-after studies, etc.)
- Intervention: Type(s) of intervention analyzed
- Comparator: What are the interventions compared to—could include multiple comparators such as no intervention, standard of care, etc.
- Outcome Type: Quantitative, qualitative, or mixed? Indicate if a meta-analysis is performed
- Outcome: Objectives analyzed in the systematic review. If a meta-analysis is done, report the results
- Risk of bias: Is any risk of bias tool used? If yes, specify the exact name.

**Objectively Summarize the Risk of Bias Findings**

- Ethical considerations: Are there any specific ethical considerations about artificial intelligence or chatbots?
- Language: What language(s) were accepted for articles included in the review?
- Chatbot Type: What technology did the chatbots use? Conversational chatbot, NLP-based chatbot, LLM-based chatbot? Try to be precise and technical.
- Reporting: Any special reporting considerations specific to chatbots?
