# Supplementary material for: Critical Appraisal Tools for Evaluating Artificial Intelligence in Clinical Studies: Scoping Review
Source: J Med Internet Res. 2025 Dec 8;27:e77110. doi: 10.2196/77110 (PMC12685289; doi:10.2196/77110)
Supplement: Multimedia Appendix 3 [file jmir-v27-e77110-s003.docx]

**Exclusions After Full-Text Screening** (59)

- SAIT: Specific artificial intelligence tools
- NSAIT: Nonspecific artificial intelligence tools
- SR-SAIT: Systematic Reviews of AI in which the studies were assessed with specific artificial intelligence tools (42). The names and references of these AI tools are included in this scoping review. (refer to PRISMA flowchart*/Identification of studies via other methods/citation searching of SR of AI studies*)
- SR-NSAIT: Systematic Reviews of AI in which the studies were assessed with nonspecific artificial intelligence tools (or traditional) 8.
- DNMIC: Does not meet inclusion criteria (**2).**
- MEC: meets exclusion criteria (**7).**

| Author | Year, DOI, reference | Title | Cause of exclusion | - SAI tools tracked (included) and NSAIT Tracked (classical, not included) |
| --- | --- | --- | --- | --- |
| De Silva, R | 2019  10.1071/PYv25n3abs  Australian Journal of Primary Health - Volume 25, Issue 3, pp. xxviii-xxix - published 2019-01-01 | Use and performance of machine learning models for type 2 diabetes prediction: | SR-SAIT | - SAIT: Luo et al (2016) - NSAIT used CHARMS TRIPOD |
| Creswell K. | 2020  [10.1177/1460458219900452](https://doi.org/10.1177/1460458219900452)  Health Informatics J. 2020 Sep;26(3):2138-2147 | Investigating the use of data-driven artificial intelligence in computerized decision support systems for health and social care | SR-NSAIT | - No new Tools - NSAIT used Cochrane RoB traditional and CASP Guides |
| Marwah, R | 2021  Journal of Medical Imaging and Radiation Oncology - Volume 65, Issue 0, pp. 52 - published 2021-01-01 | Artificial intelligence imaging applications in the differentiation of true tumor progression from treatment-related effects in brain tumors | SR-SAIT | - SAIT: CLAIM - NSAIT used: PROBAST |
| Siontis, G. C. M | 2021  10.1136/bmjhci-2021-100466  BMJ Health and Care Informatics - Volume 28, Issue 1, pp. e100466 - published 2021-01-01 | Development and validation pathways of artificial intelligence tools evaluatin randomized clinical trials | MEC | - No new tools |
| Moor, M. | 2021  10.3389/fmed.2021.607952  Frontiers in Medicine - Volume 8, Issue 0, pp. 607952 - published 2021-01-01 | Early Prediction of Sepsis in the ICU Using Machine Learning | SR-SAIT | - SAIT: QIAO |
| Farook, | 2021  10.1155/2021/6659133  Pain research & management - Volume 2021, Issue 0, pp. 6659133 - published 2021-01-01 | Machine Learning and Intelligent Diagnostics in Dental and Orofacial Pain Management: | SR-SAIT | - SAIT MI-CLAIM - NSAIT used: JBI-DTA appraisal |
| Wu, Carine | 2022  10.1186/s13244-022-01340-2  Insights into imaging - Volume 13, Issue 1, pp. 202 - published 2022-01-01 | Automatic segmentation of prostate zonal anatomy on MRI: a systematic review of the literature | SR-SAIT | - SAIT: CLAIM, - NSAIT used: QUADAS Y DISCERN |
| Sushentsev, | 2022  10.1186/s13244-022-01199-3  Insights into imaging - Volume 13, Issue 1, pp. 59 - published 2022-01-01 | Comparative performance of fully-automated and semi-automated artificial intelligence methods for the detection of clinically significant prostate cancer on MRI | SR-SAIT | - SAIT: RQS, y CLEAR - NSAIT used: QUADAS 2 |
| Bertl, M | 2022  10.3389/fpsyt.2022.923613  Frontiers in Psychiatry - Volume 13, Issue 0, pp. 923613 - published 2022-01-01 | A systematic literature review of AI-based digital decision support systems for post-traumatic stress disorder | SR-NSAIT | - No new tools |
| Evangelista, K | 2022  DOI: 10.1007/s00784-022-04742-0  Clinical oral investigations - Volume 26, Issue 12, pp. 6893-6905 - published 2022-01-01 | Accuracy of artificial intelligence for tooth extraction decision-making in orthodontics. A systematic reviews and meta-analysis | SR-SAIT | - SAIT: Schwendicke F( 2021) |
| Kao | 2022  10.1007/s11547-022-01510-8  La Radiologia medica - Volume 127, Issue 7, pp. 754-762 - published 2022-01-01 | A meta-analysis of the diagnostic test accuracy of CT-based radiomics for the prediction of COVID-19 severity | SR-SAIT | - SAIT: RQS (Radiomics quality score) - NSAIT used: QUADAS 2 |
| Hilbert, A | 2022  International Journal of Stroke - Volume 17, Issue 3, pp. 20-21 - published 2022-01-01 | ARTIFICIAL INTELLIGENCE FOR DECISION SUPPORT IN ACUTE ISCHEMIC STROKE CARE | SR-SAIT | - SAIT: MINIMAR (Minimum Information for Medical AI reporting) |
| Jia, L | 2022  0.1016/j.ejro.2022.100438  European Journal of Radiology Open - Volume 9, Issue 0, pp. 100438 - published 2022-01-01 | Artificial intelligence model on chest imaging to diagnose COVID-19 and other pneumonias: | SR-SAIT | - SAIT: RQS, CLAIM - NSAIT used: QUADAS 2 |
| Shimpi | 2022  10.3390/jpm12040614  Journal of personalized medicine - Volume 12, Issue 4, pp. - published 2022-01-01 | Development and Validation of a Non-Invasive, Chairside Oral Cavity Cancer Risk Assessment Prototype Using Machine Learning Approach | MEC (exclusion criteria) | - No new tools |
| Kodenko, M.R. | 2022  10.3390/diagnostics12123197  Diagnostics (Basel, Switzerland) - Volume 12, Issue 12, pp. - published 2022-01-01 | Diagnostic Accuracy of AI for Opportunistic Screening of Abdominal Aortic Aneurysm in CT. | SR-NSAIT | - No new tools - NSAIT used: QUADAS2 and Cochrane RoB 2 |
| Toit, C. D | 2022  10.1097/01.hjh.0000835956.81410.5e  Journal of Hypertension - Volume 40, Issue 0, pp. e78 - published 2022-01-01 | NVESTIGATING THE QUALITY OF MACHINE LEARNING RESEARCH AND REPORTING IN HYPERTENSION | SR-SAIT | - HUMANE. (*Harmonious Understanding of Machine Learning Analytics Network survey).* - J Am Heart Assoc. 2023;12:e027896. DOI: 10.1161/JAHA.122.027896 (incluido) |
| HickmanS.E. | 2022  10.1148/radiol.2021210391  Radiology - Volume 302, Issue 1, pp. 88-104 - published 2022-01-01 | Machine Learning for Workflow Applications in Screening Mammography | SR-NSAIT | - Other: QUADAS 2 |
| Kamel R. | 2022  10.1016/j.ijmedinf.2022.104758  International journal of medical informatics - Volume 162, Issue 0, pp. 104758 - published 2022-01-01 | Machine learning models for diabetes management in acute care using electronic medical records | SR-SAIT | - **CLAIM+** - NSAIT used: TRIPOD & PROBAST |
| Zhou | 2022  10.1016/j.ijmedinf.2021.104641  International journal of medical informatics - Volume 157, Issue 0, pp. 104641 - published 2022-01-01 | Machine learning predictive models for acute pancreatitis: | SR-SAIT | - **IJMEDI checklist** |
| Bradshaw T. | 2022  [2159-662X](http://www.worldcat.org/issn/2159-662X)  Journal of Nuclear Medicine August 2022, 63 (supplement 2) 2724 | Pitfalls in the development of artificial intelligence algorithms in nuclear medicine and how to avoid them | MEC (exclusion criteria, focused in algorithmit) | - No new tools |
| Chopannejad, S. | 2022  10.1055/a-1863-1589  Applied clinical informatics - Volume 13, Issue 3, pp. 720-740 - published 2022-01-01 | Predicting Major Adverse Cardiovascular Events in Acute Coronary Syndrome: A Scoping Review of Machine Learning Approaches | SR-SAIT | - SAIT: QIAO |
| Lans A. | 2022  10.1016/j.artmed.2022.102396  Artificial intelligence in medicine - Volume 132, Issue 0, pp. 102396 - published 2022-01-01 | Quality assessment of machine learning models for diagnostic imaging in orthopaedics: | SR-SAIT | - SAIT: CLAIM |
| Jayakumar  Sounderajah | 2022  10.1038/s41746-021-00544-y  npj Digital Medicine - Volume 5, Issue 1, pp. 11 - published 2022-01-27 | Quality assessment standards in artificial intelligence diagnostic accuracy systematic | SR-NSAIT | - No new tools - NSAIT: QUADAS 2 |
| Alabed, S | 202210.3389/fcvm.2022.956811 Frontiers in Cardiovascular Medicine - Volume 9, Issue 0, pp. 956811 - published 2022-01-01 | Quality of reporting in AI cardiac MRI segmentation studies | SR-SAIT | - SAIT: CLAIM |
| Assadi H. | 2022  10.3390/medicina58081087  Medicina (Kaunas, Lithuania) - Volume 58, Issue 8, pp. - published 2022-01-01 | The Role of Artificial Intelligence in Predicting Outcomes by Cardiovascular Magnetic Resonance: A Comprehensive | SR-SAIT | - SAIT: CLAIM |
| Van Lieshout | 2022  10.1111/codi.16276  Colorectal Disease - Volume 24, Issue 0, pp. 278 - published 2022-01-01 | Tools for quality assessment of technical skill in laparoscopic surgery; | MEC (exclusion criteria) | - No new tools - Note: Classical Review with a very Interesting approach |
| Zhong | 2023  10.1186/s13018-023-03863-w  Journal of orthopaedic surgery and research - Volume 18, Issue 1, pp. 414 - published 2023-01-01 | A systematic review of radiomics in giant cell tumor of bone (GCTB): the potential of analysis on individual radiomics feature for identifying genuine promising imaging biomarkers | SR-SAIT | - SAIT: CLAIM Y RQS |
| Zhang, A | 2023  10.1101/2023.11.14.23298525  medRxiv - Volume 0, Issue 0, pp. - published 2023-01-01 | ChatGPT Exhibits Gender and Racial Biases in Acute Coronary Syndrome Management | MEC (exclusion criteria) | - No new tools |
| Heston, T | 2023  10.1101/2023.11.29.23299214  medRxiv - Volume 0, Issue 0, pp. - published 2023-01-01 | ChatGPT Provides Inconsistent Risk-Stratification of Patients With Atraumatic Chest Pain | MEC (exclusion criteria) | - No tools |
| Pattathil, | 2023  10.1136/bmjhci-2023-100757  BMJ health & care informatics - Volume 30, Issue 1, pp. - published 2023-01-01 | Adherence randomized  controlled  trials using artificial intelligence in ophthalmology to CONSORT-AI guidelines: | SR-SAIT | - SAIT: CONSORT AI - Other: Cochrane RoB 2 |
| He, Xin, | 2023  10.1080/10447318.2023.2235882  International Journal of Human-Computer Interaction - Volume 0, Issue 0, pp. No-Specified - published 2023-01-01 | AI-CDSS design guidelines and practice verification. | DNMIC | - No new tool - Note. Excellent clasical review |
| Zhang | 2023  10.1016/j.ijmedinf.2023.105024  nternational journal of medical informatics - Volume 173, Issue 0, pp. 105024 - published 2023-01-01 | Artificial intelligence-based snakebite identification using snake images, snakebite wound images, and other modalities of information | SR-SAIT | - SAIT: ChAIMAI, (IJMEDI), CLAIM and MINIMNAR**.** - Note: a good comparison between CLAIM y and MINIMAR |
| Moazemi, | 2023  10.3389/fmed.2023.1109411  Frontiers in Medicine - Volume 10, Issue 0, pp. 1109411 - published 2023-01-01 | Artificial intelligence for clinical decision support for monitoring patients in cardiovascular ICUs: | SR-NSAIT | - Np new tools - Note: Use a different and sensible criteria |
| Ozkara | 2023  10.3390/cancers15020334  Cancers - Volume 15, Issue 2, pp. - published 2023-01-01 | Deep Learning for Detecting Brain Metastases on MRI: | SR-SAIT | - SAIT CLAIM - NSAIT used: QUADAS 2 |
| Sharan, | 2023  10.1016/j.ijmedinf.2023.105093  International journal of medical informatics - Volume 176, Issue 0, pp. 105093 - published 2023-01-01 | Detecting acute respiratory diseases in the pediatric population using cough sound features and machine learning: | SR-SAIT | - SAIT: ChAMAI (IJMEDI) |
| Cruz Rivera | 2023  10.26633/RPSP.2023.149  Pan American Journal of Public Health / Revista Panamericana de Salud Pública - Volume 47, Issue 0, pp. 1-17 - published 2023-01-01 | Directrices para los protocolos de ensayos clínicos de intervenciones con inteligencia artificial: la extensión SPIRIT-AI | SR-SAIT | - SAIT : **CONSORT AI, SPRIT AI** - (In spanish version) |
| Palak | 2023  10.1016/j.bspc.2023.104915  Biomedical Signal Processing and Control - Volume 85, Issue 0, pp. 104915 - published 2023-01-01 | Effect of selection bias on Automatic Colonoscopy Polyp Detection | SR-SAIT | - SAIT: CLAIM |
| Chen | 2023  10.1001/jamanetworkopen.2023.1671  JAMA network open - Volume 6, Issue 3, pp. e231671 - published 2023-01-01 | Evaluation of Risk of Bias in Neuroimaging-Based Artificial Intelligence Models for Psychiatric Diagnosis: | SR-SAIT | - SAIT: CLEAR - NSAIT used: PROBAST |
| Malik, S.2023 | 2023  10.5489/cuaj.8322  Canadian Urological Association Journal - Volume 17, Issue 11, pp. - published 2023-01-01 | Existing trends and applications of artificial intelligence in urothelial cancer: A scoping review | SR-SAIT | - SAIT: (STREAM-URO) |
| Moulaei, | 2023  10.1016/j.ijmedinf.2023.105243  International journal of medical informatics - Volume 179, Issue 0, pp. 105243 - published 2023-01-01 | Machine learning for prediction of viral hepatitis: | SR-SAIT | - SAIT: MI-CLAIM and IJMED |
| Iancu A. | 2023  10.1016/j.ijmedinf.2023.105241  International journal of medical informatics - Volume 180, Issue 0, pp. 105241 - published 2023-01-01 | Machine learning in medication prescription: | SR-SAIT | - SAIT: IJMEDI checklist |
| Dragos, H. M | 2023  10.3390/diagnostics13050857  Diagnostics - Volume 13, Issue 5, pp. 857 - published 2023-01-01 | MRI Radiomics and Predictive Models in Assessing Ischemic Stroke Outcome- | SR-SAIT | - SAIT: RQS (radiomics quality score) - NSAIT: PROBAST |
| Krepper, D | 2023  10.1016/j.jval.2023.09.2100  Value in Health - Volume 26, Issue 12, pp. S401 - published 2023-01-01 | MSR41 Machine Learning and Patient-Reported Outcomes (PROs) in Oncology: | SR-SAIT | - SAIT: CLAIM - Note: The SR is ondoing (PROSPERO) |
| Kim | 2023  10.3348/kjr.2023.1027  Korean journal of radiology - Volume 24, Issue 12, pp. 1179-1189 - published 2023-01-01 | Reporting Quality of Research Studies on AI Applications in Medical Images According to the CLAIM Guidelines in a Radiology Journal | SR-SAIT | - SAIT: CLAIM |
| Di Bidino | 2023  10.1017/S026646232300123X  International Journal of Technology Assessment in Health Care - Volume 39, Issue 0, pp. S32 - published 2023-01-01 | Scoping Meta-Review On Methods Used To Assess Artificial Intelligence-Based Medical Devices For Heart Failure | MEC | - No new tools |
| Almasan, O | 2023  10.3390/jcm12030942  Journal of Clinical Medicine - Volume 12, Issue 3, pp. 942 - published 2023-01-01 | Temporomandibular Joint Osteoarthritis Diagnosis Employing Artificial Intelligence | SR-SAIT | - SAIT: CLAIM |
| Wu, M. J | 2023  10.3389/fcvm.2023.1172451  Frontiers in Cardiovascular Medicine - Volume 10, Issue 0, pp. 1172451 - published 2023-01-01 | The diagnostic value of electrocardiogram-based machine learning in long QT syndrome | SR-SAIT | - SAIT: QUADAS AI |
| Belue M.J. | 2023  10.1016/j.jacr.2022.05.022  Journal of the American College of Radiology : JACR - Volume 20, Issue 2, pp. 134-145 - published 2023-01-01 | The Low Rate of Adherence to Checklist for Artificial Intelligence in Medical Imaging Criteria Among Published Prostate MRI Artificial Intelligence Algorithms | SR-SAIT | - SAIT: CLAIM |
| Bobowicz, M. | 2023  1268587760  European Journal of Translational and Clinical Medicine - Volume 6, Issue 0, pp. 20 - published 2023-01-01 | Trust and credibility of medical AI solutions. How FUTURE-AI guidelines can help | SAIT. Abstract of congress | - SAIT: FUTURE-AI |
| Dimov, G | 2024  10.1016/j.jaci.2023.11.261  Journal of Allergy and Clinical Immunology - Volume 153, Issue 2, pp. AB78 - published 2024-01-01 | Assessment of Quality of Anaphylaxis Information Presented By An Artificial Intelligence Bot Versus Clinical Guidelines | SR- NSAIT | - No new tools |
| Kanan, M | 2024  10.3390/cancers16030674  Cancers - Volume 16, Issue 3, pp. 674 - published 2024-01-01 | AI-Driven Models for Diagnosing and Predicting Outcomes in Lung Cancer: | SR-NSAIT | - No new tolos - NSAIT used: QUADAS 2 |
| Ndiaye, A. | 2024  10.1159/000536277  Caries research - Volume 0, Issue 0, pp. - published 2024-01-01 | Exploring the methodological approaches of studies on radiographic databases used in cariology to feed AI: | SR-SAIT | - SAIT: CLAIM and QUADAS AI - Sounderajah V 2021 |
| Chu Yihang | 2024  10.1016/j.oret.2024.01.013  Ophthalmology. Retina - Volume 0, Issue 0, pp. - published 2024-01-01 | Image Analysis-Based Machine Learning for the Diagnosis of Retinopathy of Prematurity | SR-SAIT | - SAIT: QUADAS AI |
| Kocak | 2024  10.4274/dir.2024.232604  Diagnostic and interventional radiology (Ankara, Turkey) - Volume 0, Issue 0, pp. - published 2024-01-01 | Meta-research on reporting guidelines for artificial intelligence: are authors and reviewers encouraged enough in radiology, nuclear medicine, and medical imaging journals? | SR-SAIT | - SAIT: CLAIM, ETC |
| Zrubka Z. | 2024  10.2196/47430  Journal of medical Internet research - Volume 26, Issue 0, pp. e47430 - published 2024-01-01 | The Reporting Quality of Machine Learning Studies on Pediatric Diabetes Mellitus: | SR-SAIT | - SAIT: MI-CLAIM |
| Chen D. | 2024  10.1016/j.xops.2024.100471  Ophthalmology Science - Volume 4, Issue 4, pp. 100471 - published 2024-01-01 | Transparency in Artificial Intelligence Reporting in Ophthalmology | SR-SAIT | - SAIT: CONSORT AI y MI-CLAIM |
| Yin | 12023  0.1016/j.ijmedinf.2023.105044  International journal of medical informatics - Volume 174, Issue 0, pp. 105044 - published 2023-01-01 | Deep learning for pancreatic diseases based on endoscopic ultrasound: | SR-SAIT | - SAIT: IJMEDI checklist |
| Liu, X | 1268589455  Investigative Ophthalmology and Visual Science - Volume 61, Issue 7, pp. - published 2020-01-01 | CONSORT-AI and SPIRIT-AI: New reporting guidelines for clinical trials and trial protocols for artificial intelligence interventions | DNMIC (opinion paper) | - No new tools |
| Kolbinger FR, | 2024  10.1038/s43856-024-00492-0  Communications Medicine \| (2024)4:71 | Reporting guidelines in medical artificial intelligence: a systematic review and meta-analysis | SR-SAIT | SAIT:   - Luo et al (2016) - FDA (2023) - Jones et al (2022) |

**Están todos**

ME SALEN 42 RECUPERACIONES DE 18 NUEVOS instrumentos (quitados lo srepetidos)

MI CLAIM 13

QIAO 2

RQS 4

CLEAR 2

MINIMAR 1

HUMANE 1

CLAIM 2

JIMEDI 2

QUADAS AI 2

CHAMAI 3

STEAM URO1

DISCERN ¿?

LUO 1

FDA 1

JONES 1
