# Supplementary material for: Critical Appraisal Tools for Evaluating Artificial Intelligence in Clinical Studies: Scoping Review
Source: J Med Internet Res. 2025 Dec 8;27:e77110. doi: 10.2196/77110 (PMC12685289; doi:10.2196/77110)
Supplement: Multimedia Appendix 4 [file jmir-v27-e77110-s004.docx]

**Figure 1.** PRISMA 2020 flow diagram (adapted for Scoping reviews).

**Table 1.** Tools for critical appraisal and related constructs.

The table is organized into several columns, each providing specific information:

- **Author/Year/Ref**: Lists the author(s) of the tool, the year of publication, and the reference number.
- **Name of Tool**: Specifies the name or acronym of the tool.
- **Clinical Aim**: Describes the primary focus of the tool, such as diagnosis, prognosis, treatment, or research. CA/ML/AI (Critical appraisal, machine learning, or Artificial intelligence)
- **Clinical Area/Specialty**: Indicates the medical or clinical specialty where the tool is applied (eg, radiology, oncology, dermatology).
- **Setting/Context of Use**: Provides information on the specific context or setting where the tool is used, such as diagnosis, prognosis, treatment, or social aspects like identifying algorithmic bias. ML: machine learning.
- **Design**: Describes the design of the tool, whether it is expert-based, partially collaborative, or comprehensive. The best (comprehensive) designs, which include an explicit search, Delphi consensus, and more refined elaboration with those that are not able to ascribe the entire process, are mostly in relation to the constructs designed by expert authority criteria

**Table 2.** Name of the acronym and explanation or meaning of the tools retrieved

The table presents the following information:

- Author, Year, Ref: Lists the author(s) of the tool, the year of publication, and the reference number.
- Name or Acronym: Provides the name or acronym of the critical appraisal tool.
- Development, Explanation, or Meaning: Offers a brief description or explanation of the tool, including its purpose, development, and key features.

**Table 3.** Bias and bias mitigation. The table is structured to present the following information:

- Author, Year: Lists the author(s) and the year of publication.
- Title: Provides the title of the study or paper.
- Bias Classification: Describes the types of bias identified in the study, such as algorithmic bias, data collection bias, measurement bias, and human bias.
- Bias Mitigation: Outlines the strategies or methods proposed to mitigate the identified biases, including preprocessing techniques, algorithmic adjustments, and post-processing methods.
- Comments: Offers additional insights or examples from the study, such as specific applications, datasets, or frameworks used to address bias.

**Table 4.** Chatbot assessment studies (primary research).

The table provides a detailed overview of recent studies evaluating the performance and effectiveness of various chatbot models, particularly in clinical and medical contexts.

- Author/Ref: Lists the author(s) and reference number of the study.
- Year: Indicates the year of publication.
- Topic: Describes the primary focus of the study, such as the assessment of chatbot accuracy, consistency, or decision-making in clinical settings.
- Population: Specifies the target population or context in which the chatbot was evaluated (eg, clinical questions, surgical management, or cancer screening).
- Intervention: Details the chatbot model or system being evaluated (eg, ChatGPT, GPT-4, Bing AI, or retrieval-augmented models).
- Gold Standard/Comparison: Indicates the benchmark or standard used for comparison (eg, expert responses, clinical guidelines, or other chatbot models).
- Outcome: Reports the key findings or outcomes of the study, such as accuracy, consistency, empathy, or bias.
- Type of Chatbots: Specifies the type of chatbot evaluated, such as large language models (LLMs) such as GPT-3.5, GPT-4, or retrieval-augmented models.
- Reporting: Provides additional comments or insights from the study, such as limitations, hallucinations, reproducibility issues, or ethical concerns.

**Table 5.** The table, Chatbot Assessment Studies (Primary Research), provides a detailed overview of recent studies evaluating the performance and effectiveness of various chatbot models, particularly in clinical and medical contexts. It is structured to present the following information:

- Author/Ref: Lists the author(s) and reference number of the study.
- Year: Indicates the year of publication.
- Topic: Describes the primary focus of the study, such as the assessment of chatbot accuracy, consistency, or decision-making in clinical settings.
- Studies: Specify the type of studies conducted (eg, randomized clinical trial (RCT), cohort studies, quasi-experimental studies) and include the number of participants involved in each study.
- Intervention: Provide details on the different areas of training or non-training interventions (eg, psychology, social persuasion, behavioral strategies, etc).
- Gold Standard/Comparison: Indicates the benchmark or standard used for comparison (eg, expert responses, clinical guidelines, or other chatbot models).
- Outcome: Reports the key findings or outcomes of the study, such as accuracy, consistency, empathy, or bias.
- Type of Chatbots: Specifies the type of chatbot evaluated, such as large language models (LLMs), such as GPT-3.5, GPT-4, or retrieval-augmented models.
- Reporting: Provides additional comments or insights from the study, such as limitations, hallucinations, reproducibility issues, or ethical concerns.

**Figure 2.** Types of the construct.

**Figure 3.** Clinical setting or specialty.

**Figure 4.** Constructs and year of publication as AI tools.

**Figure 5.** Constructs, year of publication, and name of tools.

**Figure 6.** Design style for each type of construct.

**Footnote**: Comprehensive designs: when they include a systematic review, Delphi consensus, and a refined elaboration. Partially collaborative design: Designs that omit parts of the process. Expert: those based on expert authority criteria.

**Figure 7.** Number of items (average) associated with each of the constructs.
