# Supplementary material for: Critical Appraisal Tools for Evaluating Artificial Intelligence in Clinical Studies: Scoping Review
Source: J Med Internet Res. 2025 Dec 8;27:e77110. doi: 10.2196/77110 (PMC12685289; doi:10.2196/77110)
Supplement: Checklist 1 [file jmir-v27-e77110-s005.docx]

| **Section/topic** | **#** | **Checklist item** | Location(s) Reported |
| --- | --- | --- | --- |
| INFORMATION SOURCES AND METHODS | | | |
| Database name | 1 | Name each database searched, stating the platform for each. | The following electronic databases were searched:  MEDLINE(R) ALL / PubMed(R) (Ovid), Embase (Ovid), APA PsycInfo (Ovid), CINAHL (EBSCOhost), IEEE Xplore |
| Multidatabase searching | 2 | If databases were searched simultaneously on a single platform, state the name of the platform, listing all of the databases searched. | Not applicable |
| Study registries | 3 | List any study registries searched. | PROSPERO (<https://www.crd.york.ac.uk/prospero/> ), OSF (<https://osf.io/>), and Research Registry (<https://www.researchregistry.com/> ). |
| Online resources and browsing | 4 | Describe any online or print source purposefully searched or browsed (e.g., tables of contents, print conference proceedings, websites), and how this was done. | *We also search in the* EQUATOR Network’s library of reporting guidelines (<https://www.equator-network.org/library/> ). Artificial intelligence OR machine learning AND reporting guidelines. |
| Citation searching | 5 | Indicate whether cited references or citing references were examined, and describe any methods used for locating cited/citing references (e.g., browsing reference lists, using a citation index, setting up email alerts for references citing included studies). | Reference lists of included studies and other relevant reviews were manually searched in order to identify any additional studies.  References about quality assessment tools of studies mentioned in systematic reviews of AI studies were identified in screening and were included in the review when they were specific AI tools (snowballing). However, the Systematic review was excluded. Otherwise, when the tools identified in these Systematic reviews were non-specific tools, both SR and tools were excluded.  References about Bias in AI in the screening selected papers were identified, and full text was assessed and included if met the inclusion criteria. |
| Contacts | 6 | Indicate whether additional studies or data were sought by contacting authors, experts, manufacturers, or others. | We contacted experts in artificial intelligence from “Medicineai” and other groups. |
| Other methods | 7 | Describe any additional information sources or search methods used. | We include some articles from our personal registries, as well as some incidental findings that were added to the structured search process. |
| SEARCH STRATEGIES | | | |
| Full search strategies | 8 | Include the search strategies for each database and information source, copied and pasted exactly as run. | **Database: Ovid MEDLINE(R) ALL / PubMed(R) <1946 to Present>  Search Strategy:** **2024 April 12** 1  exp Deep Learning/ or exp Computer Simulation/ or exp Machine Learning/  2  (deep learning or computer simulation or computer vision or machine learning).ti,ab.  3  large language model*.mp.  4  chatgpt.mp.  5  exp Artificial Intelligence/  6  (artificial adj2 intelligence).ti,ab.  7  (LLM or ML or DL or NLP or AI).mp. and ARTIFICIAL INTELLIGENCE.ti,ab.  8  exp Checklist/ or checklist*.mp.  9  reporting guideline*.ti.  10  (toolkit* or (quality adj2 tool*) or (clinical adj2 tool*) or (risk adj2 tool*)).mp.  11  (clinical adj1 (AI or artificial intelligence) adj1 Research).mp.  12  (("artificial intelligence" or IA) adj2 (stud* or research* or investigation*)).mp.  13  ("deep learning" adj2 (stud* or research* or investigation*)).mp.  14  ("large language model*" adj2 (stud* or research* or investigation*)).mp.  15  ("computer vision" adj2 (stud* or research* or investigation*)).mp.  16  ("machine learning" adj2 (stud* or research* or investigation*)).mp.  17  (reporting adj2 guideline*).mp.  18  exp Bias/  19  (risk adj2 bias).mp.  20  (reproducibility adj2 results).mp. or exp "Reproducibility of Results"/  21  (quality adj2 assessment).mp.  22  ((quality or risk) adj2 assessment).mp. 23  Risk Assessment/  24  (statistical adj2 bias).mp.  25  (critical adj2 appraisal).mp.  26  or/1-7  27  or/8-10  28  or/11-16  29  or/17-25  30  26 and 27 and 29  31  27 and 28 and 29  32  30 or 31  **Embase <1974 to 2024 April 09>**  **Database: Embase <1974 to 2024 April 09>**  **Search Strategy:**  **1**  exp artificial intelligence/  **2**  (artificial adj2 intelligence).mp. [mp=title, abstract, heading word, drug trade name, original title, device manufacturer, drug manufacturer, device trade name, keyword heading word, floating subheading word, candidate term word]  **3**  exp machine learning/  **4**  deep learning/ or "feature learning (machine learning)"/  **5**  exp computer vision/  **6**  ("machine learning" or "deep learning" or "computer vision").mp. [mp=title, abstract, heading word, drug trade name, original title, device manufacturer, drug manufacturer, device trade name, keyword heading word, floating subheading word, candidate term word]  **7**  large language model/ **8**  large language model*.mp.  **9**  exp artificial intelligence chatbot/  **10**  ChatGPT/ or chatGPT*.mp.  **11**  1 or 2 or 3 or 4 or 5 or 6 or 7 or 8 or 9 or 10  **12**  (critical adj2 appraisal).mp. [mp=title, abstract, heading word, drug trade name, original title, device manufacturer, drug manufacturer, device trade name, keyword heading word, floating subheading word, candidate term word]  **13**  (risk adj2 bias).mp. [mp=title, abstract, heading word, drug trade name, original title, device manufacturer, drug manufacturer, device trade name, keyword heading word, floating subheading word, candidate term word]  **14**  bias.mp.  **15**  (quality adj2 assessment).mp. [mp=title, abstract, heading word, drug trade name, original title, device manufacturer, drug manufacturer, device trade name, keyword heading word, floating subheading word, candidate term word] (54797)  **16**  (analysis adj2 (study or studies)).mp. [mp=title, abstract, heading word, drug trade name, original title, device manufacturer, drug manufacturer, device trade name, keyword heading word, floating subheading word, candidate term word]  **17**  risk assessment/ or statistical bias/  **18**  reproducibility/  **19**  (reproducibility adj (study or studies)).mp. [mp=title, abstract, heading word, drug trade name, original title, device manufacturer, drug manufacturer, device trade name, keyword heading word, floating subheading word, candidate term word]  **20**  validity/ or external validity/ or internal validity/  **21**  ((external or internal) adj2 validity).mp. [mp=title, abstract, heading word, drug trade name, original title, device manufacturer, drug manufacturer, device trade name, keyword heading word, floating subheading word, candidate term word]  **22**  internal consistency/  **23**  measurement accuracy/  **24**  data quality/  **25**  12 or 13 or 14 or 15 or 16 or 17 or 18 or 19 or 20 or 21 or 22 or 23 or 24  **26**  exp quality assessment tool/  **27**  (quality adj3 tool*).mp.  **28**  (checklist* or toolkit* or toolbox or (clinical adj2 tool*) or (risk adj2 tool*)).mp.  **29**  guideline*.mp.  **30**  26 or 27 or 28 or 29  **31**  11 and 25 and 30  **32**  31 not ((exp animal/ or nonhuman/) not exp human/)  **33**  limit 32 to "remove medline records"  **Database: APA PsycInfo <1806 to April Week 1 2024>**  **Search Strategy:**  **1**  exp artificial intelligence/  **2**  (artificial adj2 intelligence).mp. [mp=title, abstract, heading word, table of contents, key concepts, original title, tests & measures, mesh word]  **3**  exp machine learning/  **4**  deep learning/ or "feature learning (machine learning)"/  **5**  exp computer vision/  **6**  ("machine learning" or "deep learning" or "computer vision").mp. [mp=title, abstract, heading word, table of contents, key concepts, original title, tests & measures, mesh word]  **7**  large language model/  **8**  large language model*.mp.  **9**  exp artificial intelligence chatbot/  **10**  ChatGPT/ or chatGPT*.mp.  **11**  1 or 2 or 3 or 4 or 5 or 6 or 7 or 8 or 9 or 10  **12**  (critical adj2 appraisal).mp. [mp=title, abstract, heading word, table of contents, key concepts, original title, tests & measures, mesh word]  **13**  (risk adj2 bias).mp. [mp=title, abstract, heading word, table of contents, key concepts, original title, tests & measures, mesh word]  **14**  bias.mp.  **15**  (quality adj2 assessment).mp. [mp=title, abstract, heading word, table of contents, key concepts, original title, tests & measures, mesh word]  **16**  (analysis adj2 (study or studies)).mp. [mp=title, abstract, heading word, table of contents, key concepts, original title, tests & measures, mesh word]  **17**  risk assessment/ or statistical bias/  **18**  reproducibility/  **19**  (reproducibility adj (study or studies)).mp. [mp=title, abstract, heading word, table of contents, key concepts, original title, tests & measures, mesh word]  **20**  validity/ or external validity/ or internal validity/  **21**  ((external or internal) adj2 validity).mp. [mp=title, abstract, heading word, table of contents, key concepts, original title, tests & measures, mesh word]  **22**  internal consistency/  **23**  measurement accuracy/  **24**  data quality/  **25**  12 or 13 or 14 or 15 or 16 or 17 or 18 or 19 or 20 or 21 or 22 or 23 or 24  **26**  exp quality assessment tool/  **27**  (quality adj3 tool*).mp.  **28**  (checklist* or toolkit* or toolbox or (clinical adj2 tool*) or (risk adj2 tool*)).mp.  **29**  guideline*.mp.  **30**  26 or 27 or 28 or 29  **31**  11 and 25 and 30  **32**  limit 31 to "remove medline records"  **CINHAL (EBSCOHOST)**  S1- (MH "Deep Learning")  S2- (MH "Computer Simulation+")  S3- (MH "Machine Learning+")  S4- (MH "Artificial Intelligence+")  S5- deep learning OR computer simulation OR computer vision OR machine learning OR large language model* OR chatgpt OR artificial intelligence  S6- (LLM or ML or DL or NLP or AI)  S7- S1 OR S2 OR S3 OR S4 OR S5 OR S6  S8- (MH "Checklists")  S9- (MH "Bias (Research)+")  S10- (MH "Risk Assessment")  S11- statistic* N2 APPRAISAL  S12- CRITICAL* N2 APPRAISAL  S13- RISK N2 BIAS  S14- reproducibility N2 results  S15- S8 OR S9 OR S10 OR S11 OR S12 OR S13 OR S14  S16- (checklist* or toolkit* or (quality N2 tool*) or (clinical N2 tool*) or (risk N2 tool*))  S17- S7 AND S15 AND S16  **IEEE Xplore 2024-04-26**  ("All Metadata":ARTIFICIAL INTELLIGENCE OR "All Metadata":CHATGPT OR "All Metadata":DEEP LEARNING OR "All Metadata":MACHINE LEARNING OR "All Metadata":LARGE LANGUAJE MODEL OR "All Metadata":COMPUTER VISION) AND ("All Metadata":risk of bias OR "All Metadata":reproducibility of result* OR "All Metadata":quality assessment OR "All Metadata":risk of assessment or statistical bias OR "All Metadata":quality of risk) AND ("All Metadata":CLINICAL TOOL* OR "All Metadata":CLINICAL CHECKLIST)  The following information typology was discarded: “Conferences” |
| Limits and restrictions | 9 | Specify that no limits were used, or describe any limits or restrictions applied to a search (e.g., date or time period, language, study design) and justify their use. | Experimental non-human studies were excluded. No language limitations were used. |
| Search filters | 10 | Indicate whether published search filters were used (as originally designed or modified), and if so, cite the filter(s) used. | Filter used in Embase and PsycInfo: "remove medline records"  Filter used in IEEE Xplore: removed the following information type: “Conferences” |
| Prior work | 11 | Indicate when search strategies from other literature reviews were adapted or reused for a substantive part or all of the search, citing the previous review(s). |  |
| Updates | 12 | Report the methods used to update the search(es) (e.g., rerunning searches, email alerts). | Search in medline studies identified in registries (2024/06/12/.) |
| Dates of searches | 13 | For each search strategy, provide the date when the last search occurred. | Medline (OVID) 2024/04/12  Embase (OVID) 2024/04/10  PsycInfo (OVID) 2024/04/11  CINAHL (EBSCOHOST) 2024/04/15  IEEE XPLORE 2024/04/26  PROSPERO 2024/04/10  EQUATOR Network’s library of reporting guidelines 2024/05/10  OSF 2024/04/18  Research Registry (2024/06/12) |
| PEER REVIEW | | | |
| Peer review | 14 | Describe any search peer review process. | YES: The search strategies were reviewed by a second health sciences librarian (MMUC & EAZ) |
| MANAGING RECORDS | | | |
| Total Records | 15 |  | **Records identified from Databases and registers** **(n = 4392 )**   1. MEDLINE=920 2. EMBASE=1558 3. PSYINFO=92 4. CINAHL=1561 5. IEEE=257 6. Registers (OSF 1, Research Registry1 PROSPERO2) = 4   **Records identified via other methods**: 58  EQUATOR Network’s library of reporting guidelines: n= 12.  Organizations & experts n =4.  Records identified form snowballing from Systematic reviews of Specific  artificial intelligence tools (SR-SAIT) in full text review. N=42. |
| Deduplication | 16 | Describe the processes and any software used to deduplicate records from multiple database searches and other information sources. | We used two reference managers: Zotero as main tool and Endnote to manager some databases.  We used Rayyan systematic reviews software ([www.Rayyan.ai](http://www.Rayyan.ai) ) with Artificial intelligence to automatically identify duplicates and after we deleted duplicates in a supervised process.  We also used Rayyan systematic reviews software for the 1^st^ phase of the screening process (title + abstracts) and for the 2^nd^ phase of the screening (full text screening). |
